# l-Isoleucine-Schiff Base Copper(II) Coordination Polymers: Crystal Structure, Spectroscopic, Hirshfeld Surface, and DFT Analyses

**DOI:** 10.1021/acsomega.3c02878

**Published:** 2023-06-28

**Authors:** Iván F. Chavez-Urias, Luis E. López-González, Damian F. Plascencia-Martínez, Juventino J. García, Marcos Flores-Alamo, Rocío Sugich-Miranda, Felipe Medrano, Lorenzo A. Picos-Corrales, Karla-Alejandra López-Gastélum, Enrique F. Velázquez-Contreras, Fernando Rocha-Alonzo

**Affiliations:** † Departamento de Investigación en Polímeros y Materiales, Universidad de Sonora, Calle Rosales y Blvd. Luis Encinas s/n, Col. Centro, Hermosillo, Sonora 83000, México; ‡ Centro de Investigación Científica y de Educación Superior de Ensenada, Carretera Tijuana-Ensenada 3918, Zona Playitas, Ensenada, Baja California 22860, México; § Facultad de Química, Universidad Nacional Autónoma de México, Circuito Exterior Cd. Universitaria, Coyoacán, Ciudad de México, Ciudad de México 04510, México; ∥ Departamento de Ciencias Químico-Biológicas, Universidad de Sonora, Calle Rosales y Blvd. Luis Encinas s/n, Col. Centro, Hermosillo, Sonora 83000, México; ⊥ Facultad de Ingeniería Culiacán, Universidad Autónoma de Sinaloa, Ciudad Universitaria, Culiacán, Sinaloa 80013, México; # Centro de Investigación en Alimentación y Desarrollo, Carretera a Ejido La Victoria Km 0.6, Hermosillo, Sonora 83304, México

## Abstract

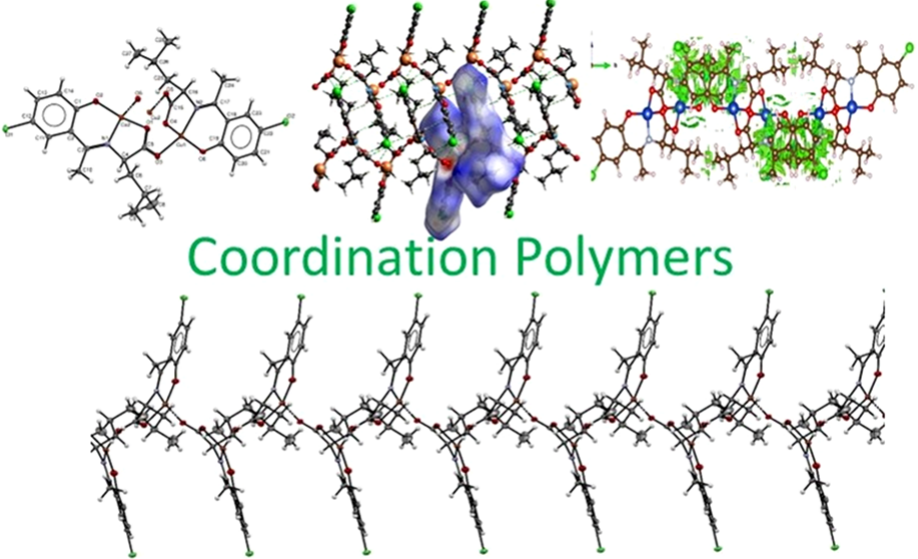

A new copper(II) coordination polymer was synthesized from the l-isoleucine-Schiff base and characterized by elemental analysis, Fourier transform infrared (FT-IR) spectroscopy, ultraviolet–visible (UV–vis) spectroscopy, single-crystal X-ray diffraction (XRD) analysis, electronic paramagnetic resonance, and thermogravimetric analysis. XRD analysis confirmed the square planar coordination geometry of metallic centers and a zipper-like polymer structure. Vibrational, electronic, and paramagnetic spectroscopies and thermal analysis were consistent with the crystal structure. A Hirshfeld surface (HS) and density functional theory (DFT) analyses were employed to gain additional insight into interactions responsible for complex packing. The quantitative examination of two-dimensional (2D) fingerprint plots revealed, among other van der Waals forces, the dominating participation of H···H and H···Cl interactions in the molecular packing. The use of computational methods provided great help in detailing the supramolecular interactions occurring in the crystal, which were mainly van der Waals attractions. The electronic transition analysis helped corroborate the electronic transitions observed experimentally in the absorption spectrum. The frequency and vibrational mode analysis gave a deeper insight into the characterization of the CuL_CL_ complex.

## 1 Introduction

In recent years, oligonuclear metal complexes have been used as building blocks for synthesizing organometallic frameworks and coordination polymers. Coordination polymers have become increasingly important in materials science due to coordination bonds and non-covalent interactions (NCIs) to generate self-assemblies of various dimensions with esthetic values and countless applications that paved the way for supramolecular chemistry/crystal engineering.^1^ The structural variety, exciting properties, and potential applications in catalysis, luminescence, gas adsorption, and magnetic materials have attracted researchers to these compounds.^2,3^ The indispensable goal of complex coordination formation in crystal engineering is to find exciting properties and unique privileged bonding features that require a reasonable choice of ligands and metal precursors, so it is a huge challenge in coordination chemistry to discover new polymeric structures with a great diversity of transition metals.^4−9^ Among the various transition metals, copper(II) complexes are gaining more importance because they are relevant in biological fields;^10,11^ these metallic complexes exhibit rich coordination chemistry, and, in conjunction with their abundance and low cost, have stimulated researchers to use copper-based compounds in different types of chemical processes.^6^ There is a wide variety of copper(II) coordination polymers reported in the literature, where the design of the materials is mainly based on covalent metal–ligand interactions.^12−16^ In this work, a new copper coordination polymer is presented, where, in addition to these types of interaction, non-covalent interactions are observed that give it a different supramolecular structure and, therefore, a diversity of new applications to be explored. Coordination polymers with Schiff base ligands are an essential class of coordination compounds with unique properties and applications in various fields; these materials have potential applications in gas storage, separation, and catalysis.^4^ So the design and synthesis of copper(II) complexes of Schiff bases have been an attractive area of research.^17,18^

In the present work, we report the synthesis of one new copper(II) complex CuLCl, where the LCl stands for a polydentate Schiff base ligand derived from l-isoleucine, which, being a naturally occurring amino acid, makes this class of compounds green, low cost, and easy to synthesize. X-ray crystallography has been used to characterize the new complex, and the structure can be described as a polymeric complex bound by different types of NCIs. Non-covalent interactions are potent tools for crystal engineering and supramolecular building architectures of coordination compounds that may be discrete or polymeric.^19^ Here, crystal structure analysis breaks down the complex’s minute structural features, while further quantifying the intermolecular interactions through Hirshfeld surface analysis and density functional theory (DFT) analysis.^20−22^

## 2 Results and Discussion

### 2.1 Description of the Crystal Structure

**Figure 1:**
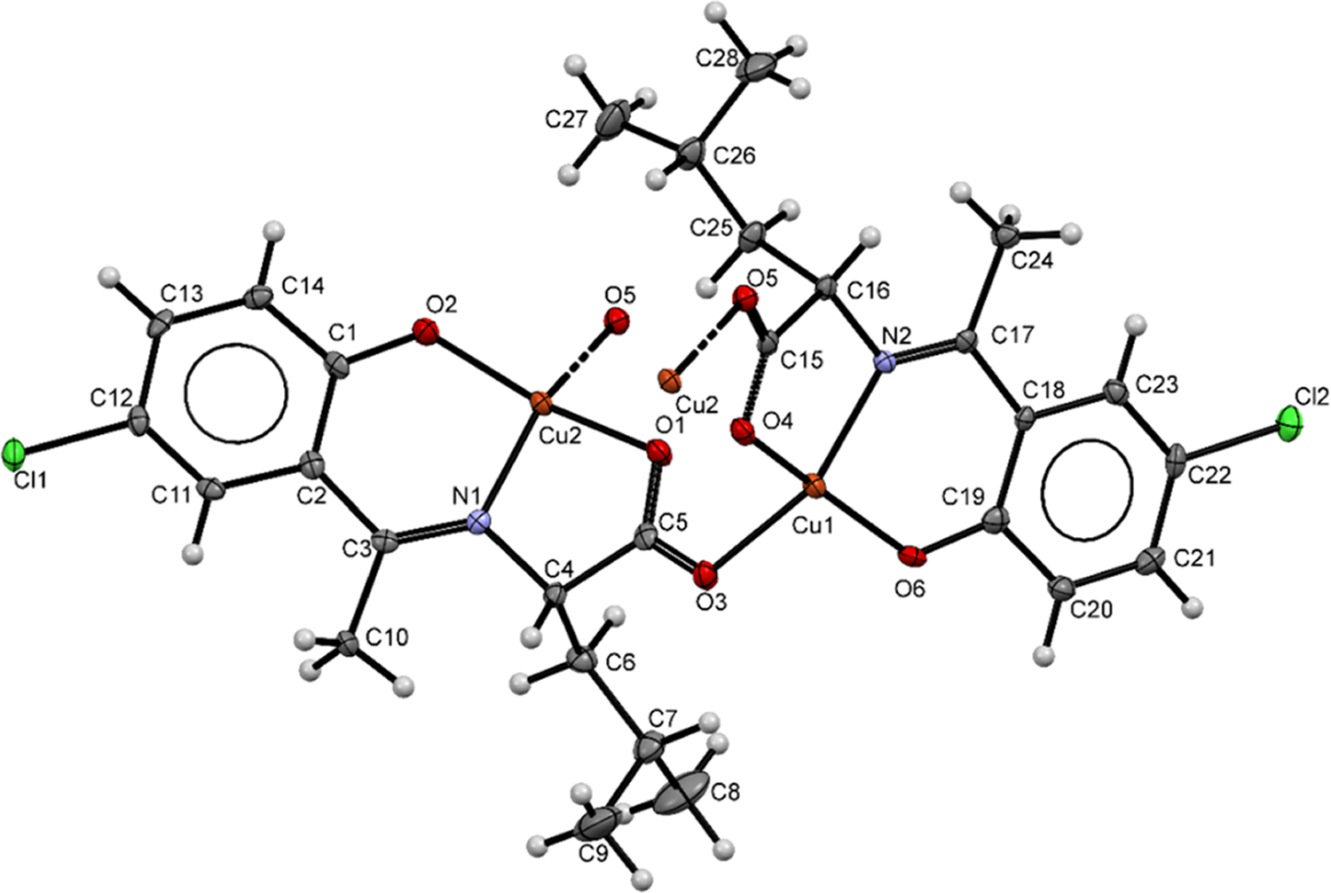
ORTEP representation (drawn with 50% thermal ellipsoid) showing atomic labeling of the asymmetric unit of the compound.

**Figure 2:**
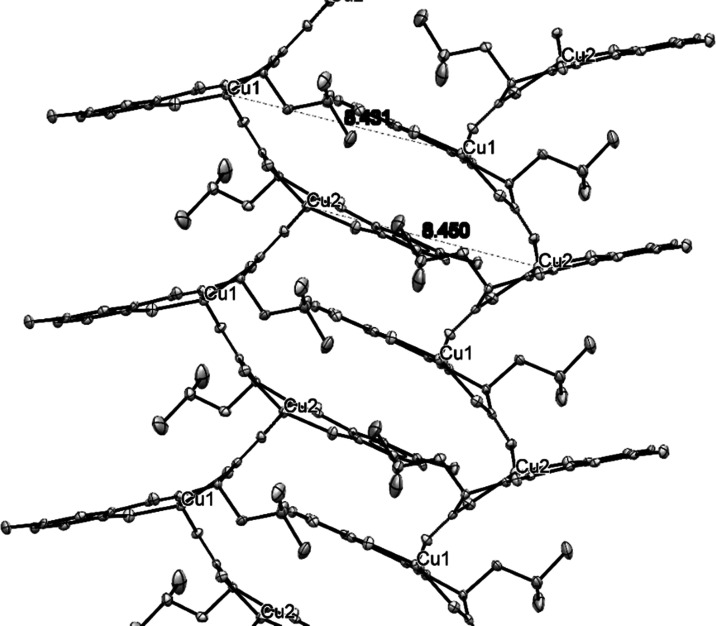
Two adjacent chains of complex CuL_Cl_ fit to form a zipper-like structure.

The compound crystallizes in the orthorhombic *P*2_1_2_1_2_1_ space group. A perspective view of the complex with an atom labeling scheme is shown in Figure 1. Selected bond lengths and angles are given in Table 1. The asymmetric unit of this polymeric polymorph houses two crystallographically distinct molecules, with both the copper(II) centers (Cu1 and Cu2) disposing of in distorted square planar topology. Interestingly, a one-dimensional polymeric chain constituting both Cu1 and Cu2 units is built by the end-on bridging of the second oxygen of the carboxylate group (Figure 2). The geometry of the metal ions when tracked using the τ_4_ index returns a value of 0.35 and 0.33 for Cu1 and Cu2 respectively, which reveals a subtle distortion from the ideal square planar geometry; the τ_4_ index value of unity indicates an ideal tetrahedral geometry while a value of zero indicates an ideal square planar geometry τ_4_ = [360 – (α + β)]/141°, where α and β (in °) are the two most numerous angles around the central metal in the complex.^23^ The Schiff base ligand coordinates in a tridentate form with the metal atom using its phenolic oxygen, azomethine nitrogen, and carboxylate oxygen atoms, forming two fused metallocycles, one of six-membered Cg(2) (comprised of Cu(1), O(6), C(19), C(18), C(17), and N(2) atoms) and other five-membered Cg(3) (comprised of Cu(1), N(2), C(16), C(15), and O(4) atoms).

**Table 1:** Selected Bond Lengths and Angles for CuL_Cl_

bond lengths (Å)		bond angles (°)	
Cu(1)–O(6)	1.852(3)	O(6)–Cu(1)–N(2)	95.59(13)
Cu(1)–N(2)	1.933(3)	O(6)–Cu(1)–O(4)	155.81(12)
Cu(1)–O(4)	1.974(3)	N(2)–Cu(1)–O(4)	85.08(13)
Cu(1)–O(3)	1.980(3)	O(6)–Cu(1)–O(3)	95.51(12)
Cu(2)–O(2)	1.855(3)	N(2)–Cu(1)–O(3)	154.56(13)
Cu(2)–N(1)	1.934(3)	O(4)–Cu(1)–O(3)	93.92(12)
Cu(2)–O(1)	1.967(3)	O(2)–Cu(2)–N(1)	95.71(14)
Cu(2)–O(5)#1	1.980(3)	O(2)–Cu(2)–O(1)	156.32(12)
		N(1)–Cu(2)–O(1)	85.07(13)
		O(2)–Cu(2)–O(5)#1	94.14(12)
		N(1)–Cu(2)–O(5)#1	156.07(13)
		O(1)–Cu(2)–O(5)#1	94.47(12)

The bond distances in the complex follow the following order: metal–carboxylate oxygen (1.980(3) Å) > metal–other carboxylate oxygen (1.974(3) and 1.967(3) Å) > metal–imino nitrogen (1.933(3) Å) > metal–phenoxo oxygen (1.852(3) and 1.855(3) Å). The non-bonded distances of Cu1 with the symmetrically related copper centers (Cu2 (*x*, −1 + *y*, −1 + *z*)) along this chain are 4.768 and 4.799 Å, respectively. With interchain Cu–Cu distances of 8.431 and 8.450 Å, this complex is much more closely packed, as revealed by the comparatively lower interchain Cu–Cu distances with other polymeric complexes of a similar structure.^1^

### 2.2 Supramolecular Aspects in the Complex

**Figure 3:**
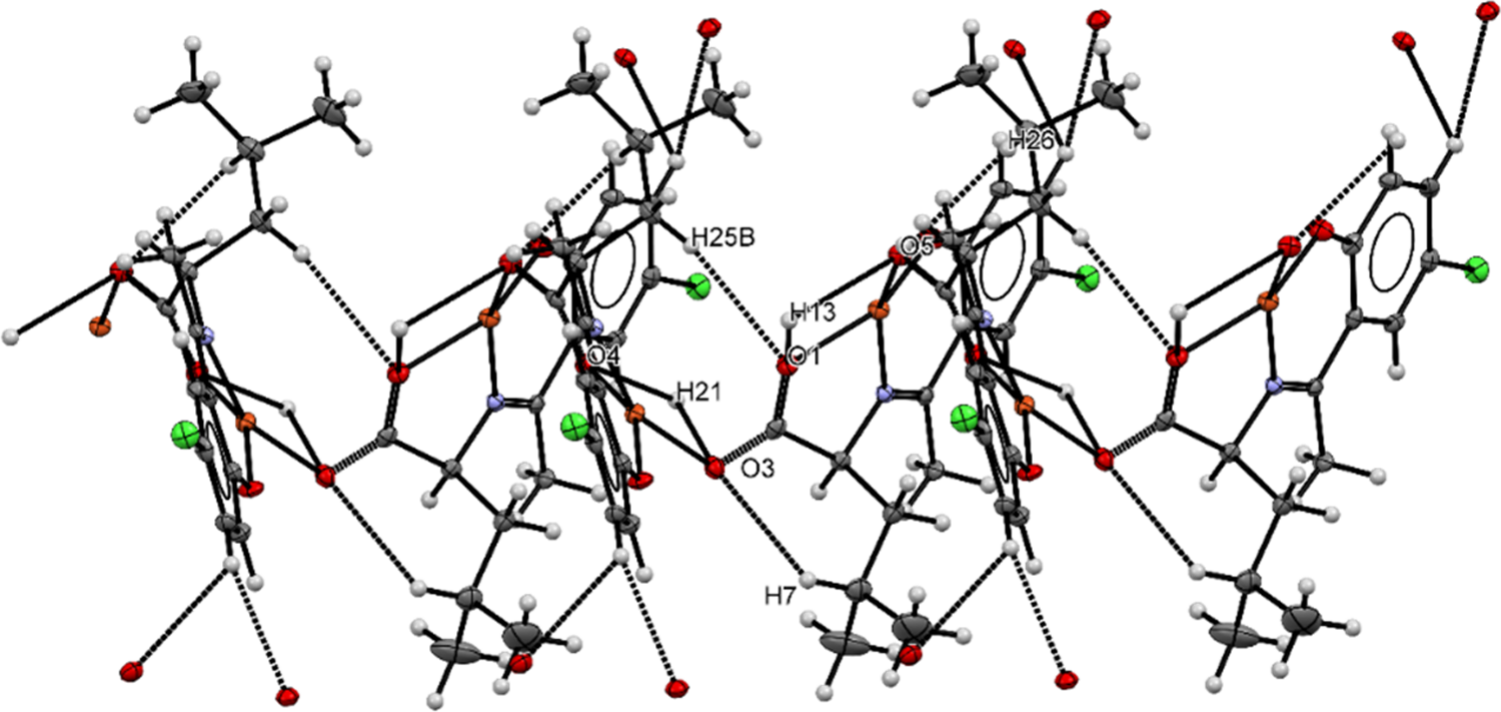
Hydrogen bonding (dashed lines) in complex CuL_Cl_.

In the complex, different types of non-classical intermolecular hydrogen bonding interactions are found (Figure 3). Two hydrogen atoms, H7 borne by the C7 atom and H21 borne by the C21 atom form an intermolecular hydrogen bonding interaction with oxygen atom O3. The same hydrogen H21 atom forms another intermolecular hydrogen bond with the oxygen atom O4 borne by the C15 atom. Carboxylate oxygen, O1, is also engaged in intermolecular hydrogen bonding with hydrogen atom H13 borne by C13 and H25B borne by C25. Another carboxylate oxygen, O5, engages an intermolecular hydrogen bonding with two hydrogen atoms, H13 borne by the C13 atom and H26 borne by the C26 atom.

### 2.3 Hirshfeld Surface Analysis

**Figure 4:**
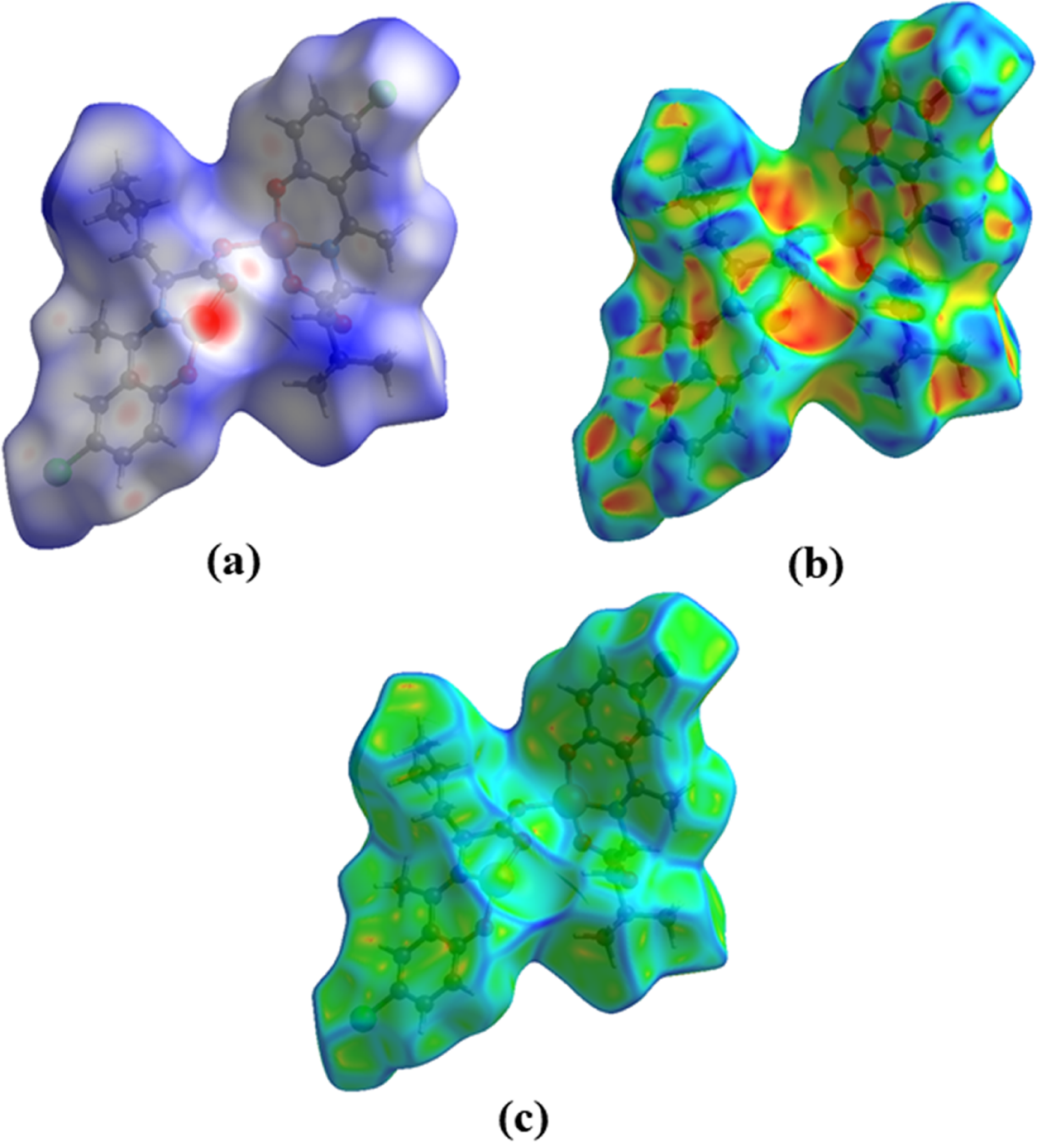
Hirshfeld surface of complex CuL_Cl_ mapped with (a) dnorm, (b) shape index, and (c) curvedness.

The asymmetric unit of the compound was used to generate the Hirshfeld surface (HS) of the complex. Maps of the Hirshfeld surface, shape index, and curvedness were generated based on the crystallographic information file (CIF) using the CrystalExplorer program (Figure 4).^24,25^ HSs mapped with dnorm, shape index, and curvedness are represented in a transparent manner to aid easy visualization (Figure S1). Hirshfeld surfaces enable the visualization of intermolecular interactions and indicate the relative strength of the interactions by different colors and color intensity. A measure of globularity (*G*) and sphericity (Ω) of the surfaces gave a value of 0.640 and 0–178, respectively, which shows that the surface is deviating from the spherical shape and a degree of anisotropy is present.^26,27^

**Figure 5:**
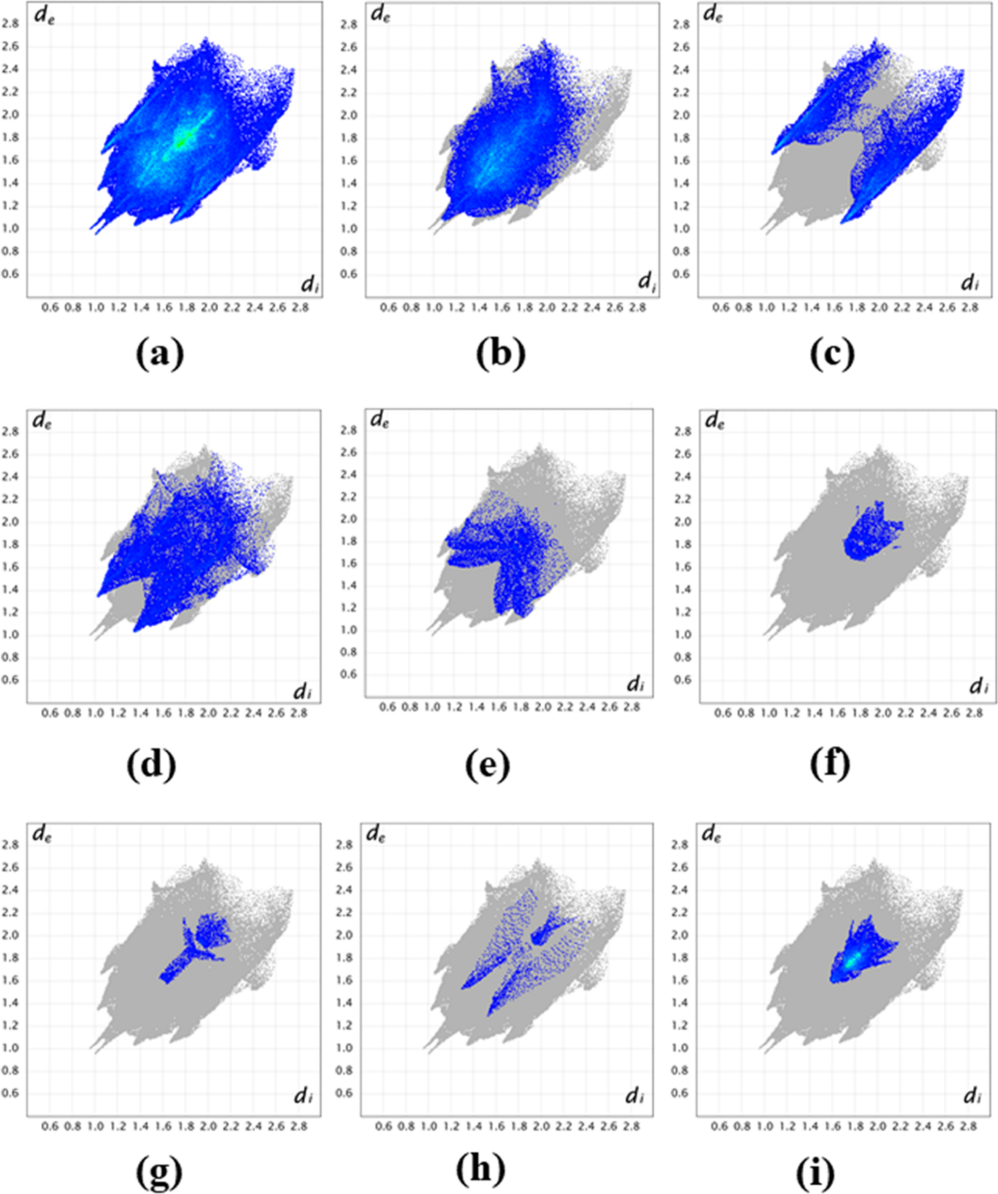
2D molecular fingerprint plots for CuL_Cl_, showing (a) all NCI, and plots specifically illustrating the (b) H···H, (c) Cl···H/H···Cl, (d) H···O/O···H, (e) C···H/H···C, (f) C···N/N···C, (g) C···O/O···C, (h) O···O, and (j) C···C interactions.

**Figure 6:**
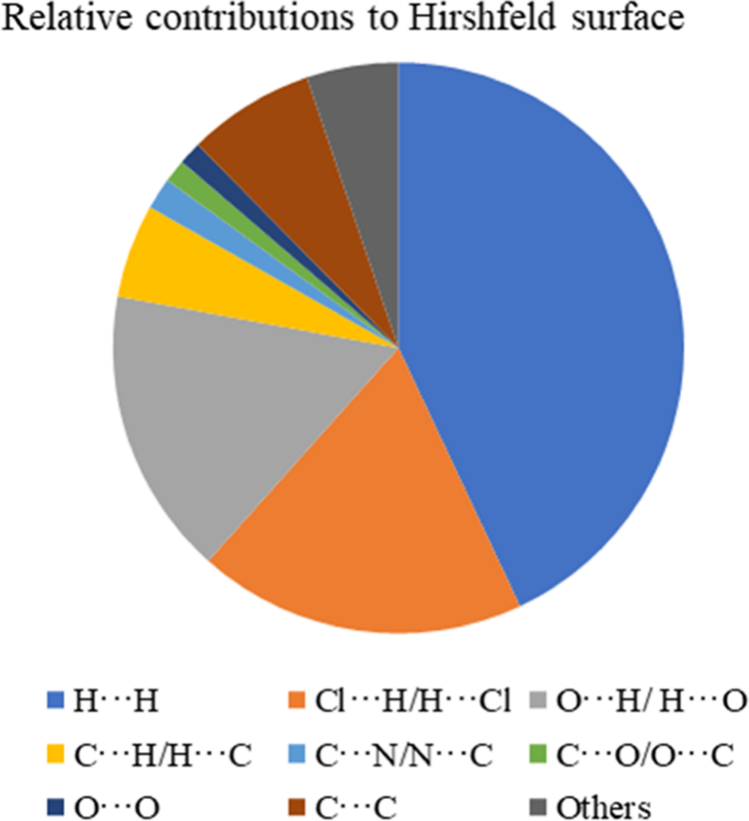
Relative contributions from short atom contacts to the Hirshfeld surface.

Two-dimensional (2D) fingerprint plots of Hirshfeld surfaces for the complex are shown in Figure 5. Analysis of the 2D fingerprint plots reveals that the significant contribution corresponding to 43% of the surface is due to H···H contacts; the next highest contribution to the total Hirshfeld surface is from Cl···H/H···Cl interactions (18.6%) identified as two sharp spikes, which are seen on the top left and bottom right panels. The remaining contacts, i.e., O···H/H···O, C···H/H···C, C···N/N···C, C···O/O···C, and O···O comprise 16.3, 5.3, 1.8, 1.3, and 1.3%, respectively, for crystal structure stabilization. Finally, C···C interactions are indicated by the green area in the central section of the 2D fingerprint plots and evidence π–π interactions (C···C, 7.2%). Relative contributions of all interactions in the complex are shown in Figure 6.

### 2.4 IR Spectra

The tentative assignments of the significant infrared (IR) spectral bands of the Schiff bases and its copper(II) complex are presented in Table 2 and Figure S2. A comparison of the IR spectra of the ligand and metal complex shows that significant variations have occurred in the characteristic frequencies upon complexation. The spectrum of Schiff base ligand exhibits bands at 3423 and 1615 cm^–1^, which can be assigned to ν(O–H) and ν(C=O) ν(C=N) stretching vibrations, respectively. The absence of the ν(O–H) stretching mode in the spectra of the complex suggests that the ligand loses this proton on complexation.^28^ The shift in ν(C=O) and ν(C=N) stretching frequencies was observed from 1615 cm^–1^ in the ligand to 1598 cm^–1^ for complex supports coordination via carboxylate oxygen and azomethine nitrogen.^29,30^

**Table 2:** Infrared Spectral Data of the Schiff Base Ligand and Complex (cm^–1^)

compound	ν(OH)	ν(C=O)	ν(C=N)	ν_asym_(COO^–^)	ν_sym_(COO^–^)
L_Cl_	3423	1615	1615	1464	1395
CuL_Cl_		1598	1598	1452	1313

### 2.5 Thermogravimetric Studies

Thermogravimetric (TG) analyses of the complex gave information concerning the thermal stability and the nature of water molecules in the complex. The thermal decomposition of complex CuL_Cl_ is summarized in Table 3 and the thermogram is presented in Figure S3. The thermal stability of the complex was investigated by TG/derivative TG (TG/DTG) measurements, and the curves were obtained at a heating rate of 5 °C min^–1^ in an air atmosphere over the temperature range of 25–800 °C. The complex is stable up to 109 °C since there is no weight loss observed. The thermal decomposition pattern can be ascribed to three weight losses. The first weight loss of 5.0% (calcd 4.9%) was observed between 109 and 214 °C indicating the removal of two water molecules from the compound. The second stage of decomposition starts at 214 °C and ends at 282 °C. It corresponds to the loss of the amino acid fraction of the complex (weight loss of 37% (calcd 36%)) from the complex. The third weight loss was observed between 282 and 475 °C, corresponding to the removal of another organic moiety, 5-chloro-2-hydroxyacetophenone leaving CuO as a metallic residue.^31,32^

**Table 3:** Thermal Analytical Data for the Complex CuL_Cl_

				mass loss (%)		
molecular formula	Stage	TGA temp. (°C)	DGT temp. (°C)	found	calcd	asgmt.
C_28_H_36_Cl_2_Cu_2_N_2_O_8_	1	109–242		5	4.9	2H_2_O
	2	242–282	264	37	36.1	
	3	282–475	294	38		

### 2.6 EDX-SEM

Energy-dispersive X-ray (EDX) is used to identify the elemental composition of the synthesized compounds. We recognize the chemical composition, such as Cu metal ions, using the EDX technique. We also identified the synthesized coordination polymer’s elemental composition of C, O, Cl, and N (Table S1 and Figure S4). Scanning electron microscopy (SEM) is popularly applied to determine the surface morphology. We performed a SEM experiment on our prepared complex, shown in Figure S5. The SEM figures in the complex directly exhibited the morphology of distributed overlapping sheets, in agreement with other complexes reported in the literature.^33^

### 2.7 Electronic Spectra

The electronic spectra of the compounds were recorded in the solution state after dissolving in methanol. The ultraviolet–visible (UV–visible) spectra of the new Schiff base ligand and the copper(II) complex have two bands; one centered at around 42,000 cm^–1^ and another at around 25,000 cm^–1^. These bands are assigned to π → π* and *n* → π* transitions of the phenyl rings and the imine moiety, respectively.^34,35^ In the chelate, absorption in the visible region (15,480 cm^–1^) range is attributable to d–d transitions. For square planar complexes with a d*_x_*_–*y*_^22^ ground state, three transitions are possible viz, d*_x_*_–*y*_^22^ → d*_xy_*, d*_x_*_–*y*_^22^ → d*_z_*^2^, and d*_x_*_–*y*_^22^ → d*_xz_*, and d*_yz_* (^2^B_2g_ ← ^2^B_1g_, ^2^A_1g_ ← ^2^B_1g_, and ^2^E_g_ ← ^2^B_1g_).^36,37^ However, due to the minimal energy difference between the d orbitals, each transition cannot be distinguished by its energy. Hence, it is not very easy to resolve the three bands into separate components.^38,39^ The electronic spectral data of the compounds are given in Table 4 and Figure S6.

**Table 4:** Electronic Spectral Data (cm^–1^) of the Compounds

compound	π–π*	*n*–π*	d–d
L_Cl_	45,662	26,525	
CuL_Cl_	39,682	27,624	15,480

### 2.8 Electron Paramagnetic Resonance Spectra

**Figure 7:**
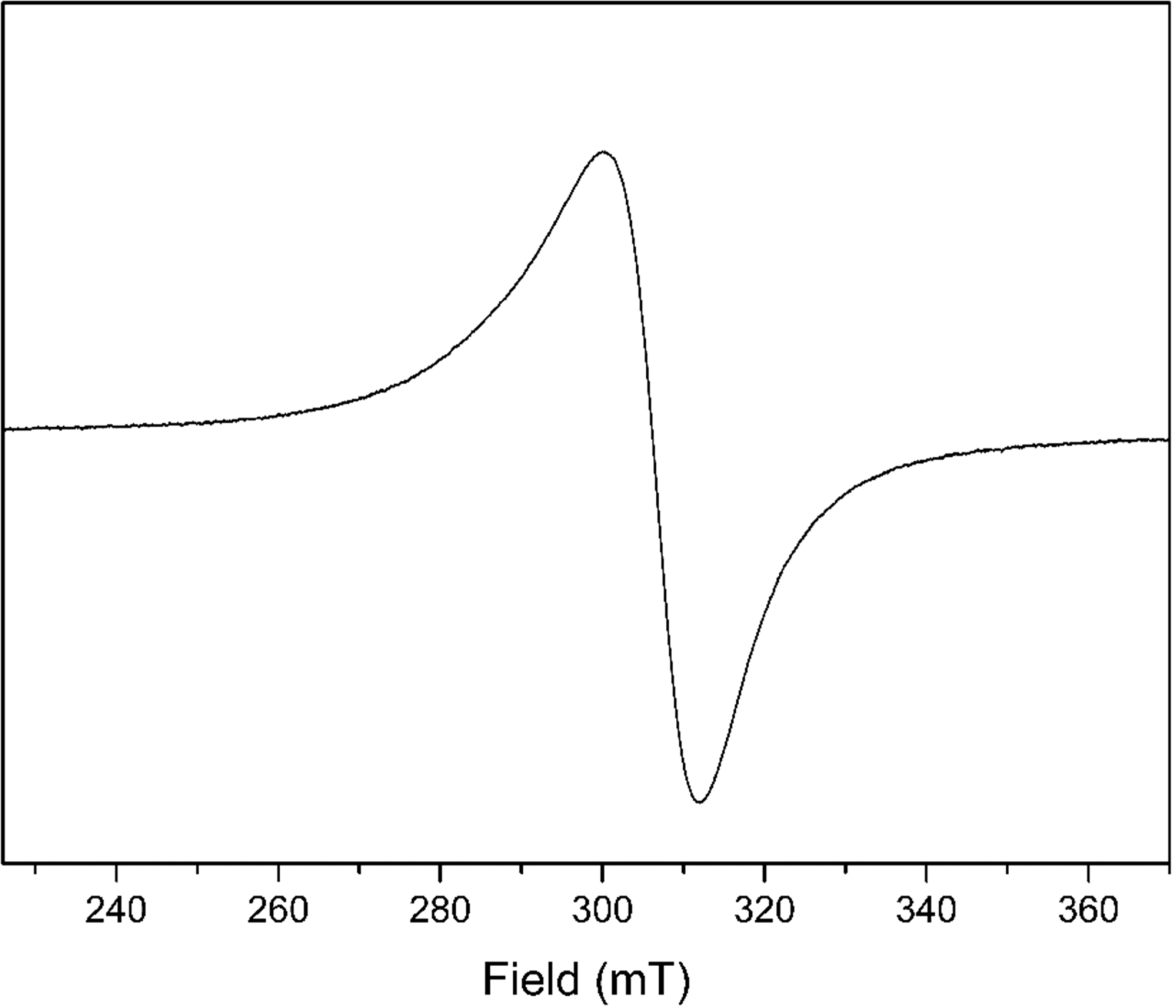
EPR spectra of complex CuL_Cl_ in the polycrystalline form at room temperature.

**Figure 8:**
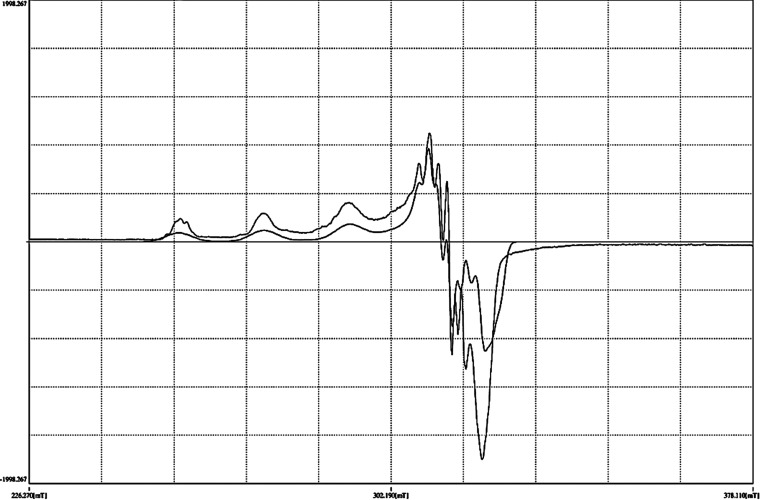
EPR spectra of complex CuL_Cl_ in the solution state at 77 K.

The electron paramagnetic resonance (EPR) spectra of the complex were recorded in polycrystalline and solution states (Figures 7 and 8). The EPR spectrum of complex CuL_Cl_ in a polycrystalline state exhibited a typical isotropic behavior with only one broad signal at *g*_iso_ = 2.110, which generally arises from extensive dipolar broadening and enhanced spin-lattice relaxation.^40^ A 77 K EPR spectrum in the solution state has axial features and four well-resolved hyperfine lines in the parallel region with *g*_||_ = 2.270 and *g*_⊥_ = 2.056. Table 5 shows the EPR spectral assignments.

**Table 5:** EPR Spectral Assignments of the Complex CuL_Cl_ in the Polycrystalline State at 298 K and Solution at 77 K

polycrystalline state (298 K)				methanol solution (77 K)					
*g*_*iso*_	*g*_||_	*g*_⊥_	*G*	*g*_||_	*g*_⊥_	*g*_av_	*A*_||_^a^	*A*_⊥_^a^	*A*_av_^a^
2.110				2.270	2.056	2.1273	166	16	66.0

a Values in 10^–4^ cm^–1^.

The EPR parameters *g*_||_, *g*_⊥_, *g*_av_, *A*_||_ (Cu), and *A*_⊥_ (Cu) incorporated with the energies of d–d transition were used to evaluate the bonding parameters α^2^, β^2^, and γ^2^, which are considered to be the measure of the covalency of in-plane σ-bonds, in-plane π-bonds, and out-of-plane π-bonds, respectively. The value of in-plane σ bonding parameter α^2^ was estimated from the expression.^41,42^
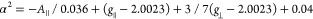
1

The following simplified expression was used to calculate the bonding parameters.^43,44^
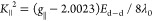
2
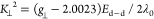
3
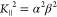
4
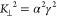
5where *K*_||_ and *K*_⊥_ are orbital reduction factors and λ_0_ represents the one electron spin–orbit coupling constant with a value of −828 cm^–1^ for the copper(II) d^9^ system.^1^ Hathaway^45^ pointed out that *K*_||_ ≈ *K*_⊥_ ≈ 0.77, for plane π-bonding, *K*_||_ < *K*_⊥_ and for out-of-plane π-bonding, *K*_⊥_ < *K*_||_, in both complexes it is observed that *K*_⊥_ < *K*_||_, which indicates the presence of significant out-of-plane π-bonding. The value of α^2^ determines the nature of the metal–ligand bond; α^2^ is unity for a purely ionic bond and is completely covalent if it is 0.5.^34^ For complex CuL_Cl_ it was found to be 0.7918; both lie between 0.5 and 1, which implies that the nature of the metal–ligand bond is partially ionic and partially covalent. Another parameter, *f*, index of tetragonal distortion, can be calculated as *f* = *g*_||_/*A*_||_ (cm), whose value may differ from 105 to 135 for small to extreme distortion and depends on the nature of the coordinated atom.^38^ In the complex, the value falls in the 136–137 cm range, suggesting a minor distortion for the copper(II) center. The EPR bonding parameters of the copper(II) complex are shown in Table 6.

**Table 6:** EPR Bonding Parameters of CuL_Cl_

α^2^	β^2^	γ^2^	*K*_||_^2^	*K*_⊥_^2^	*f*^a^
0.7918	0.7901	0.6339	0.6256	0.5019	136

a Expressed in units of cm.

### 2.9 Non-Covalent Interaction Analysis

Two computational methods were used to examine the NCI in the complex crystal. First, a plane-wave pseudopotential calculation was carried out on the periodic structure using the projector augmented wave (PAW) method^46^ implemented in the Quantum ESPRESSO^47^ with plane-wave and density cutoff values of 35 and 280 Ry, respectively, and a single γ *k*-point grid. The NCI plots^48,49^ were obtained using the critic2 program.^50^ The calculations provide a qualitative indication of the nature of the interactions between molecules in a crystal through the visualization of the gradient isosurface based on the electron density derivatives obtained from the wavefunction calculation. The results are manifested in a three-color scheme, that is, blue, red, and green, for attractive, repulsive, and weakly attractive interactions, respectively.

**Figure 9:**
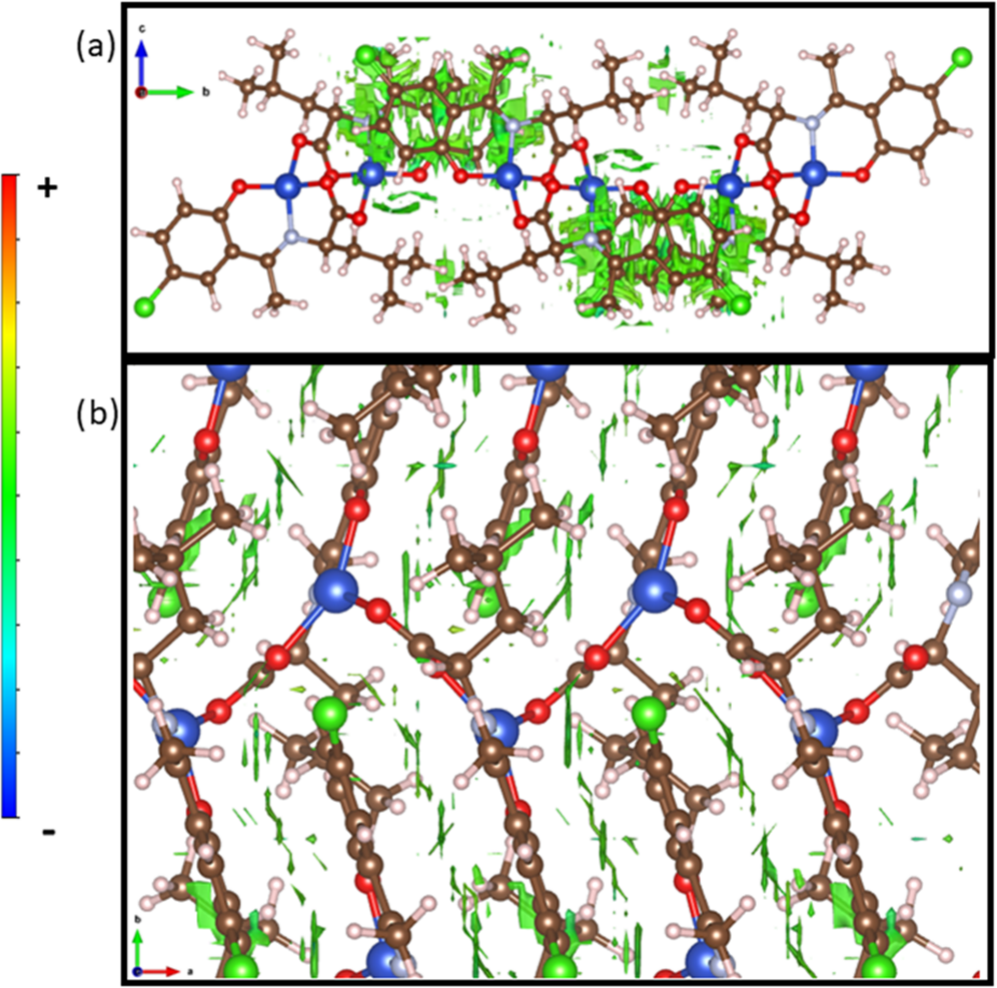
Images of the non-covalent interaction plot (reduced density gradient isosurfaces) (Iso value 0.05) for the crystal structure of the CuL_Cl_ metal complex. (a) Lateral view and (b) top view. Color scale indicates the sign(λ_2_)ρ value with blue as highly negative and red as highly positive. Green regions indicate weakly attractive interactions. Cu is shown in blue, C in brown, H in white, O in red, N in gray, and Cl in green.

Figure 9 shows the reduced density gradient (RDG) colored by density values. The results revealed a giant van de Waals attractions area between the stacked layers of the complex monomer, as shown in Figure 9a. Two major attraction regions show the corresponding symmetry from the crystal structure. Each region shows two parts of interest as follows: first, the attraction between the nitrogen atom and the carbon atoms from the aromatic ring from the contiguous monomer layer shaped like a donut isosurface. On the other hand, the offset observed between adjacent monomer layers of phenyl and chelate rings from the complex favored van der Waals interactions; this can be appreciated as the discontinuous attraction part is mainly observed between carbon atoms. Figure 9b shows the interlayer van der Waals attraction domains revealed by the RDG. The periodic stacking of van der Waals attractions observed has a cumulative effect, which may act as a stabilization component facilitating supramolecular polymer crystal formation.

### 2.10 Electronic Transitions and Complex Reactivity

**Figure 10:**
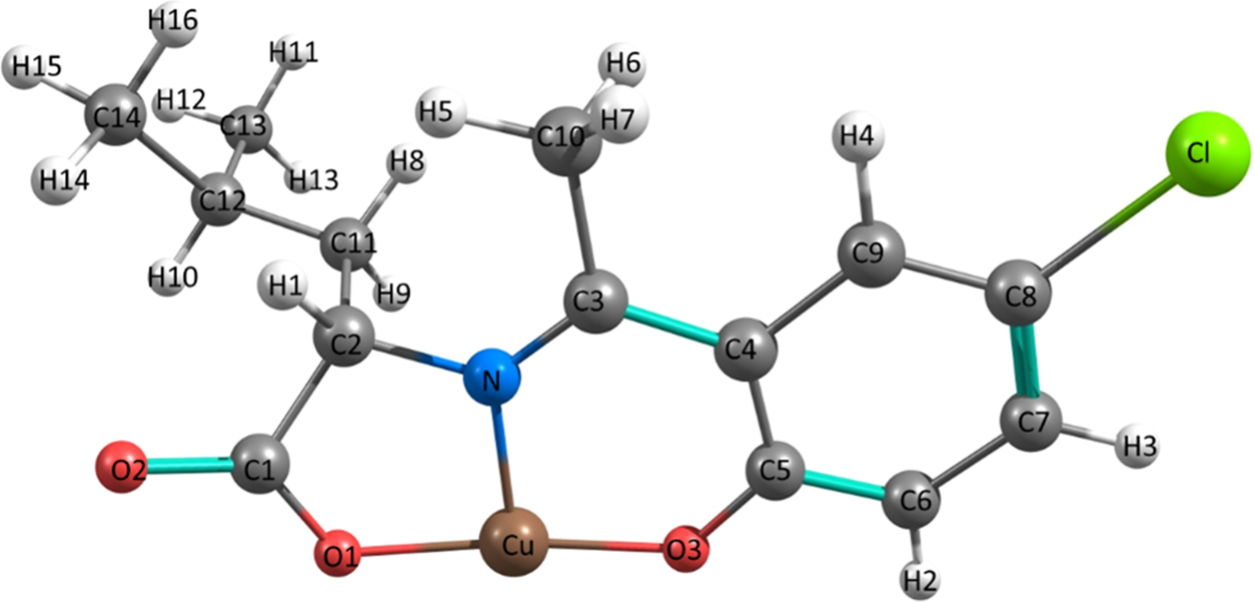
Optimized geometry for the CuL_Cl_ complex monomer using DFT (UM06-2x/LANL2DZ). Cu is shown in brown, C in gray, H in white, O in red, N in blue, and Cl in green.

Complete geometry optimization of the single-molecule CuL_Cl_ complex was performed by DFT UM06-2x functional employing the effective core potential (ECP) LANL2DZ basis set was performed using the Gaussian09^51^ software. The resulting optimized structure with the corresponding atom identification is shown in Figure 10. To obtain primary insight into the nucleophilic (Nu^–^) and electrophilic (E^+^) reactive sites of the CuL_Cl_ complex, we carried out mapping molecular electrostatic potential (MESP)^52,53^ (Figure S7), regions in red denote the negative electrostatic potential or the Nu^+^ sites, while blue color indicates the positive or electrophilic regions of the complex, this plot showed a highly nucleophilic region attributed to the oxygen atoms, while the density related to copper atoms was electrophilic. These results are consistent with the formation of a supramolecular polymer system observed in crystallographic analysis.

On the other hand, natural bond orbital (NBO) calculations were performed to study orbital effects focusing on the second-order perturbation analysis since it is convenient to characterize donor–acceptor orbital interactions. The results for the CuL_Cl_ complex, specifically for interactions corresponding to Cu are presented in Table S2. Second-order interaction energy corresponding to nitrogen and oxygen atom lone pair to the copper antibonding lone pair resulted in high values (higher than 18 kcal/mol), which indicated a very strong interaction between the ligand and metal, these results corroborate the nature of metal–ligand bonding determined by the α^2^ value in EPR experiments.

**Figure 11:**
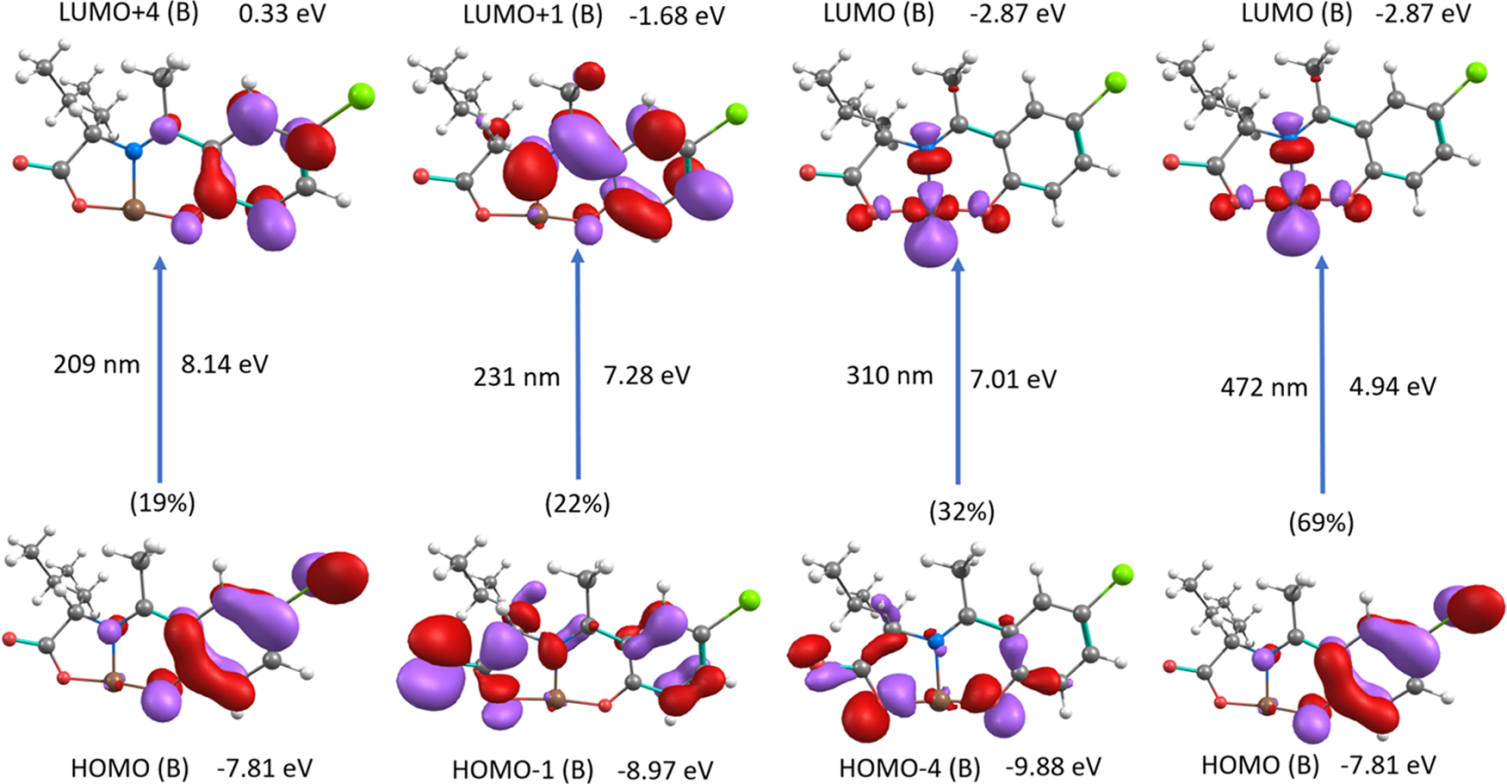
Molecular orbital distribution isosurfaces (value 0.05) for the calculated electronic transitions of the CuL_Cl_ complex. Energy values calculated for each molecular orbital, the energy differences, and the calculated absorption wavelength are shown for each transition.

To obtain insight into the spectral properties and the electronic structure of the investigated complex, time-dependent density functional theory (TD-DFT) calculations on the optimized structure were performed. The spectrum of the complex calculated by TD-UM06-2x/LANL2DZ in a vacuum is given in the supplementary material (Figure S8), and its electronic transitions are presented in Table S3. The calculated d–d excitation lengths, energies of other excitation transitions (*f* > 0.0002), and their assignments are shown in Table S3. The simulated spectrum of the complex in a vacuum agrees with the observed absorption spectrum. Four predominant electronic transitions (highest percentage of contribution) are observed in the UV region of the complex: at 209, 231, 310, and 472 nm corresponding to H(β) → L + 4(β), H – 1(β) → L + 1(β), H – 4(β) → L(β), H(β) → L + 1(β) (H: highest occupied molecular orbital (HOMO) and L: lowest unoccupied molecular orbital (LUMO)). The molecular orbital isosurfaces of the ground and excited state for each transition are shown in Figure 11. The results for 217 show that this transition takes place in the aromatic ring of the complex and is a π–π* transition. For 231 nm majoritarian transition, the HOMO – 1 shows mainly π type molecular orbital shape over the region of the carbonyl group, a nitrogen atom, and an aromatic ring, while LUMO + 1 distribution is similar, resulting in a π–π* transition too. On the other hand, in the transition occurring at 310, the HOMO – 4 shape is mainly of π character. At the same time, the LUMO is a d orbital shape with the electron density accumulation at the copper atom, resulting in a ligand-to-metal charge transfer LMCT transition. At 472 nm, the transition corresponds mainly to the HOMO → LOMO with a low oscillator strength (0.0002), the shape of the ground and excited state orbitals shows mainly a π character with density regions over the aromatic ring. In contrast, the excited state mainly shows a d orbital shape with density accumulation over the copper atom; this indicates that this transition corresponds to a ligand-to-metal charge transfer transition (LMCT).

Low oscillator strength transition (0.0003) was observed at 761 nm, in which significant contribution resulted in H-26(β) → L(β), the corresponding ground state and excited orbital isosurfaces mainly show that the characteristic shape of the d orbital in copper is a d–d electronic transition.

### 2.11 Vibrational Spectra

Theoretical vibrational spectra have been computed by the available M06-2x exchange–correlation functional DFT method. As expected, the calculated harmonic frequencies are overestimated compared to the experiment.^54^ This is mainly caused by harmonic approximation and the incomplete treatment of electron correlation in these calculations.^55^ To aid comparison between the predicted and observed frequencies, we employed the correction factor for the M06-2x functional with the LANL2DZ basis for high and low frequencies based on the report of Zhang and co-workers in 2016.^56^ The resulting calculated vibrational spectra of the complex compared to the experimental one are presented in Figure S9. The summary of the vibrational frequencies and the assignment of their vibrational modes are shown in Tables S4 and S5. Where each frequency has been scaled with the above-described scaling factor. The vibrational mode assignment was made by comparing the computational results to the experimental spectra. According to the figure, the bands observed at 2996 and at 2967 cm^–1^ correspond to the C–H stretching of C–H (C14–[H14, H15, H16]) and (C14–[H15, H16], C2–H1, C11–[H8, H9], C13–H11, H13) in an antisymmetric manner, which in the calculated spectrum resulted in scaled values of 3004 and 2979 cm^–1,^ respectively. The bands at 2896 and 2879 cm^–1^ correspond to C–H stretching. The calculated wavenumber for these vibrational modes resulted in 2894.19 and 2883.26 cm^–1^ corresponding to a symmetric stretching of C13–[H11, H12, H13] and C14–[H14, H15, H16], and C11–[H8, H9], respectively.

The most intense band in the spectrum corresponds to C=O stretching experimentally appearing at 1612 cm^–1^, while the calculated value appeared at 1645 cm^–1^ corresponding to C1=O2 stretching. The contiguous intense band at 1583 cm^–1^ in the experimental spectrum was assigned, relating it to the calculated results, where this band appeared at 1608.97 cm^–1^, corresponding mainly to C3–N, C5–O3, C4–C9, and C6–C7 symmetric stretching.

The assignment of the complete spectrum of vibrational modes is detailed in Tables S4 and S5 where the rest of the vibrational modes are described. The main IR signals of the experimental spectrum were compared to the calculated IR frequencies. In the region between 3900 and 1080 cm^–3^ (Table S4) there was a difference in the position between experimental C–H_a_ stretches, while in the experiment these bands were observed at 2996 and 2967 cm^–3^ in the calculated scaled frequencies these were shifted by almost 10 cm^–1^ to higher wavenumber values. On the other hand, signals corresponding to C–H_s_ appeared almost at the same wavenumber position in the calculated spectrum with respect to experimental results with overestimation shifts lower than ±5 cm^–1^. The carbonyl group stretching signal resulted in a 33 cm^–1^ shift with respect to the experimental result. Signals from C–O and C–N stretching also appeared to shift at around 10 cm^–1^ with respect to the experiment. Overall the overestimation of the IR signals attributed to no intermolecular interaction was considered since the molecule was modeled in a vacuum, however, the global assignment as well as the predicted bands match the experimental results.

## 3 Conclusions

In this study, we have successfully synthesized a new copper(II) coordination complex, which bears a Schiff base-amino acid ligand. The X-ray structure revealed the formation of a coordination polymer, which adopted a zipper-like design. The crystal structure in the solid state displayed various non-covalent supramolecular interactions endorsed by the Hirshfeld surface and 2D fingerprint analyses. Herein, the interesting H---H and H---Cl contacts are mainly observed in the molecular packing. The use of computational methods provided great help in detailing the interactions occurring in the crystal, which were primarily van der Waals attractions. The NBO analysis revealed strong donor–acceptor orbital interactions between oxygen and copper and nitrogen and copper. These calculations were consistent with EPR results for O–Cu and N–Cu strong bonding nature. The electronic transition analysis helped to corroborate and understand the observed electronic absorption spectrum. The frequency and vibrational mode analysis gave deeper insight into the characterization of the complex allowing a precise assignment of the experimental spectrum. The evidenced supramolecular interactions experimentally and theoretically contribute to understanding the solid-state behavior of copper(II) coordination complexes.

## 4 Materials and Methods

### 4.1 Preparation of the Copper(II) Complex

All chemicals and solvents were purchased from Sigma Aldrich Company and used as received without further purification. l-Isoleucine (98%), 5-chloro-2-hydroxyacetophenone (99%), CuSO_4_·5H_2_O (98%), CH_3_ONa (95%), and CH_3_OH (99%).

The copper(II) complex was prepared using a one-pot synthesis strategy; the first step was the synthesis of the Schiff base ligand derived from the amino acid l-isoleucine and the ketone 5-chloro-2-hydroxyacetophenone (L_Cl_) and the second step was the formation of the copper(II) complex (CuL_Cl_).

L_Cl_ was synthesized by refluxing 0.131 g (1 mmol) of l-isoleucine in MeOH with 0.170 g (1 mmol) of 5-chloro-2-hydroxyacetophenone in methanol for 24 h. For the synthesis of the complexes, methanolic ligand solutions obtained in the previous step were used, CuSO_4_·5H_2_O (250 mg, 1 mmol) in methanol was added with constant stirring, and the product was heated under reflux overnight; the mixture was cooled to room temperature and filtered to remove any precipitate; the resulting green colored solution was allowed to stand for slow evaporation at room temperature. Green needle-like crystals suitable for single-crystal XRD (SCXRD) were obtained from the de mother liquor.

CuL_Cl_: Yield: 83%; m.p. 250–251 °C. Anal calc. for (C_28_H_32_Cl_2_Cu_2_N_2_O_6_)·H_2_O (726.53): C, 46.29; H, 4.99; N, 3.86. Found: C, 46.09; H, 4.75; N, 4.01%.

### 4.2 Physical Measurements

Elemental analyses were carried out using a Thermo Scientific Flash 2000 analyzer. Elemental content and morphology of the CuLCl complex were characterized by scanning electron microscopy using a Hitachi SE-SEM 5500 equipped with an energy-dispersive X-ray analysis system. Infrared (IR) spectra were obtained using KBr pellets (solid samples) on a PerkinElmer Frontier FT-IR/FIR spectrometer in a range of 4000–500 cm^–1^. Electronic spectra were recorded in methanol on a PerkinElmer Lambda 20 UV–vis spectrophotometer from 200 to 1100 nm. The thermogravimetric measurement (TGA) was carried out using a TGA Q5000 from TA Instruments, with an airflow rate of 10 mL min^–1^ and a heating rate of 5 °C min^–1^ from 25 to 800 °C. The EPR spectra of polycrystalline samples at 298 K and solution at 77 K were recorded in quartz tubes with a Joel JES-TE300 spectrometer equipped with a cylindrical cavity (TE011 mode) operating at the X band frequency (9.4 GHz) at 100 KHz field modulation.

### 4.3 Crystallographic Data Collection and Refinement

A suitable single crystal of compound CuL_Cl_, was mounted on a glass fiber; crystallographic data were recorded with an Oxford Diffraction Gemini “A” diffractometer with a CCD area detector at 130 K, with lMoKa = 0.71073. Unit cell parameters were determined with a set of three runs of 15 frames (1° in w). The double-pass method of scanning was used to exclude any noise.^57^ The collected frames were integrated by using an orientation matrix determined from narrow frame scans. Final cell constants were determined by global refinement; collected data were corrected for absorbance by using analytical and numerical absorption correction using a multifaceted crystal model based on expressions based on the Laue symmetry with equivalent reflections.^58^ Structure solution and refinement were carried out with the SHELXS-2018^59^ and SHELXL-2018^60^ packages. WinGX v2020.2^61^ software was used to prepare the material for publication. Full-matrix least-squares refinement was carried out by minimizing (*F*_o_^2^ – *F*_c_^2^)^2^. All non-hydrogen atoms were refined anisotropically. H atoms attached to C atoms were placed in geometrically idealized positions and refined as riding on their parent atoms, with C–H = 0.95–0.99 Å and with *U*_iso_(H) = 1.2*U*_eq_(C) for aromatic and methylene groups and *U*_iso_(H) = 1.5*U*_eq_(C) for the methyl group. Attempts made to model the solvent molecule were not successful; the SQUEEZE^62^ option in PLATON indicated that there was a solvent cavity of 67 Å^3^. In the final cycles of refinement, this contribution of 16 electrons to the electron density was removed from the observed data. For electron density, the *F*(000) value of the molecular weight and the formula are given without considering the results obtained with SQUEEZE. Crystallographic data for all complexes are presented in Table 7. The crystallographic data for the structure reported in this paper have been deposited with the Cambridge Crystallographic Data Centre as supplementary publication no. CCDC 2247330. Copies of the data can be obtained free of charge by application to CCDC, 12 Union Road, Cambridge, CB2 1EZ, U.K. (fax: (+44) 1223-336-033, e-mail: deposit@ccdc.cam.ac.uk).

**Table 7:** Crystal Data and Structure Refinement for CuL_Cl_

identification code	CuL_Cl_
empirical formula	C_28_H_32_Cl_2_Cu_2_ N_2_O_6_
formula weight	690.53
temperature	130(2) K
wavelength	0.71073 Å
crystal system	orthorhombic
space group	*P*2_1_2_1_2_1_
unit cell dimensions	*a* = 7.1573(4) Å, α = 90°
	*b* = 16.6893(11) Å, β = 90°
	*c* = 24.1380(15) Å, γ = 90°
volume	2883.3(3) Å^3^
*Z*	4
density (calculated)	1.591 Mg/m^3^
absorption coefficient	1.705 mm^–1^
*F*(000)	1416
crystal size	0.570 × 0.150 × 0.110 mm^3^
θ range for data collection	3.518 to 29.573°
index ranges	–9 ≤ *h* ≤ 6, –13 ≤ *k* ≤ 21, –33 ≤ *l* ≤ 33
reflections collected	9355
independent reflections	6166 [*R*(int) = 0.0270]
completeness to θ = 25.242°	99.6%
absorption correction	analytical
max. and min. transmission	0.830 and 0.701
refinement method	full-matrix least-squares on *F*^2^
data/restraints/parameters	6166/0/367
goodness-of-fit on *F*^2^	1.041
final *R* indices [*I* > 2σ(*I*)]	*R*1 = 0.0349, *wR*2 = 0.0755
*R* indices (all data)	*R*1 = 0.0448, *wR*2 = 0.0817
absolute structure parameter	0.005(10)
extinction coefficient	n/a
largest diff. peak and hole	0.390 and −0.632 e·Å^–3^

### 4.4 Hirshfeld Surface Analysis

Hirshfeld surfaces^24,63^ and the associated two-dimensional (2D) fingerprint plots^64,65^ were calculated using CrystalExplorer 17.5 software.^66^ The intermolecular contacts present in the crystals were mapped using normalized contact distance dnorm, which is defined in terms of *d*_i_ and *d*_e_ and van der Waals radii (rvdW) of atoms by the following equation.
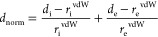
6where *d*_i_ and *d*_e_ represent the distance from the Hirshfeld surface to the nearest nucleus inside and outside the surface, and *r*_i_^vdW^ and *r*_e_^vdW^ are the van der Waals radii of the atoms. The value of *d*_norm_ is negative or positive depending on intermolecular contacts being shorter or longer than the van der Waals separations. This equation was visualized by employing a red–white–blue color code. Red represents intermolecular contacts closer than the sum of van der Waals radii, whereas blue represents regions devoid of close contacts. White color displays the contacts around the sum of van der Waals radii. Similarly, using fingerprint plots, the set of points in 2D space portrays the overall contribution of intermolecular interactions. For a given crystal structure and set of spherical atomic electron densities, the Hirshfeld surface is unique, and thus it suggests the possibility of gaining additional insight into the intermolecular interactions of molecular crystals.

### 4.5 Computational Methods

We performed first-principles calculations in the framework of periodic boundary conditions density functional theory (DFT). The Quantum espresso simulation package^47^ was employed, which uses plane-wave pseudopotentials. For the simulations, we used the cells and structures as resolved by X-ray diffraction experiments, yielding residual forces acting on each atom below 0.01 eV Å^–1^, enough to guarantee fully converged results. The Brillouin zone was modeled as a 2 × 2 × 1 *k*-point Monkhorts–Pack grid.^67^ For periodic boundary conditions calculations on the complex crystal model, the exchange–correlation energy was treated by the generalized gradient approximation (GGA) by means of the Perdew–Burke–Ernzerhof (PBE)^68^ functional. The ion–electron interactions within each atom were modeled using plane augmented wave (PAW) pseudopotentials and a plane wave basis set with a kinetic energy cutoff of 35 Ry and a charge density cutoff of 280 Ry. The dispersion correction was treated under the scheme of Grimme DFT-D3.^69,70^

To study the NCI, reduced density gradient calculations were carried out using the CRITIC2^50^* code. The NCI analysis provides an index based on the electron density and its derivatives that enable the identification of non-covalent interactions.^71^ The NCI index is based on a 2D plot of the reduced density gradient, *S*, and the electron density, ρ, as described in the following equation.^48^
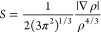
7When a weak inter- or intra-molecular interaction is present, there is a crucial change in the reduced gradient between the interacting atoms, producing density crucial points between interacting fragments. Local minima appear in *S*(ρ) associated with each critical point. Since the behavior of *S* at low densities is dominated by ρ, *S* tends to deviate except in the regions around a density critical point, where ∇ρ dominates and *S* is zero. When we search for the points in the real space responsible for this feature, the non-covalent region evidently appears as a colored isosurface.^48^

To study the electronic structure of the metal complex and to have a deeper insight into the vibrational spectrum properties of the copper complex monomer, DFT calculations were carried out using Gaussian09.^51^ The investigated compound is an open-shell system (d^9^ electron configuration of copper(II) cation); this requires unrestricted DFT methods to calculate the electronic structure. The optimized geometry, harmonic frequencies, and electronic states, as well as the UV–vis absorption spectrum, were calculated using the unrestricted Minesota exchange–correlation functional UM06-2x^72^ and the effective core potential (ECP) of Hady and Wadt LANL2DZ basis set.^73^ The starting geometry for optimization was constructed based on the crystallographic data without any geometry restrictions. The calculated ground state for the isolated complex is doublet ^2^A. The electronic spectra were calculated using the TD-DFT approach,^74^ as implemented in the Gaussian09^51^ code considering the complex optimized geometry in a vacuum.

## Supporting Information Available

Intermolecular hydrogen bonding interactions in l-HCl-IleCu, viewing the same short contacts in the Hirshfeld surface mapped with dnorm (Figure S1); infrared spectra of the ligand l-HCl-Ile and complex CuL_Cl_ (Figure S2); thermogravimetric (TG/DTG) curves of complex CuL_Cl_ (Figure S3); representative EDX profile for complex CuL_Cl_ (Figure S4); representative different SEM images of complex CuL_Cl_ (Figure S5); UV–vis spectra of ligand and complex CuL_Cl_ (Figure S6); map of molecular electrostatic potential (MESP), regions in red indicate negative electrostatic potential (ESP) and blue denotes positive ESP (Figure S7); comparison between experimental and calculated UV–vis spectra (Figure S8); experimental and calculated by the UM06-2x/LANL2DZ method (bottom) IR spectra (Figure S9); EDX analysis of weight (%) contribution of elements (Table S1); natural bond orbital analysis, donor and acceptor NBOs for interactions with Cu with indication of the second-order interaction energy E^(2)^ and donor and acceptor orbitals for complex CuL_Cl_ (Table S2); electronic transitions, their wavelength major contributions and their percentage calculated by TD-DFT M06-2X/LANL2DZ (Table S3); experimental IR and theoretical frequencies (ω, cm^–1^), infrared intensities (*I*_IR_, km mol^–1^) for the CuL_Cl_ complex in the range of 3900–1080 cm^–1^ (Table S4); experimental IR, theoretical frequencies (ω, cm^–1^), and infrared intensities (*I*_IR_, km mol^–1^) for the CuL_Cl_ complex in the range of 1080–480 cm^–1^ (Table S5); atomic coordinates (×10^4^) and equivalent isotropic displacement parameters (Å^2^ × 103) for CuL_Cl_ (Table S6); *U*(eq) is defined as one-third of the trace of the orthogonalized *U^ij^* tensor; bond lengths [Å] and angles [°] for CuL_Cl_ (Table S7); anisotropic displacement parameters (Å^2^ × 103) for CuL_Cl_ (Table S8), and the anisotropic displacement factor exponent takes the form: −2*p*^2^[*h*^2^*a**^2^*U*^11^ +··· + 2*hka***b***U*^12^] (PDF)
