# Supplementary material for: l-Isoleucine-Schiff Base Copper(II) Coordination Polymers: Crystal Structure, Spectroscopic, Hirshfeld Surface, and DFT Analyses
Source: ACS Omega. 2023 Jun 28;8(27):24601–14. doi: 10.1021/acsomega.3c02878 (PMC10339395; doi:10.1021/acsomega.3c02878)
Supplement: Supplementary file 1 — ao3c02878_si_001.pdf [file ao3c02878_si_001.pdf]

L-isoleucine-Schiff base copper(II) coordination polymer: Crystal Structure, Spectroscopic, Hirshfeld Surface, and DFT Analyses

Iván F. Chavez-Urias,[1] Luis E. López-González,[2] Damian F. Plascencia-Martínez,[1] Juventino J. García,[3] Marcos Flores-Alamo,[3] Rocío Sugich-Miranda,[4] Felipe Medrano,[4] Lorenzo A. Picos-Corrales,[5] Karla-Alejandra López-Gastélum,[4,6]* Enrique F. Velázquez-Contreras,[1] Fernando Rocha-Alonzo[4]*

1 Departamento de Investigación en Polímeros y Materiales, Universidad de Sonora, Calle Rosales y Blvd. Luis Encinas s/n, Col. Centro, Hermosillo, Sonora 83000, México.

2 Centro de Investigación Científica y de Educación Superior de Ensenada, Baja California. Carretera Tijuana-Ensenada 3918, Zona Playitas, 22860 Ensenada, B.C.

3 Facultad de Química, Universidad Nacional Autónoma de México, Circuito Exterior Cd. Universitaria, Coyoacán, Ciudad de México, Ciudad de México 04510, México.

4 Departamento de Ciencias Químico-Biológicas, Universidad de Sonora, Calle Rosales y Blvd. Luis Encinas s/n, Col. Centro, Hermosillo, Sonora 83000, México.

5 Facultad de Ingeniería Culiacán, Universidad Autónoma de Sinaloa, Ciudad Universitaria, Culiacán, Sinaloa 80013, México.

6 Centro de Investigación en Alimentación y Desarrollo, Carretera a Ejido La Victoria Km 0.6, Hermosillo Sonora 83304, México.

*Correspondence: karla.lopezgastelum@unison.mx; fernando.rochaalonzo@unison.mx

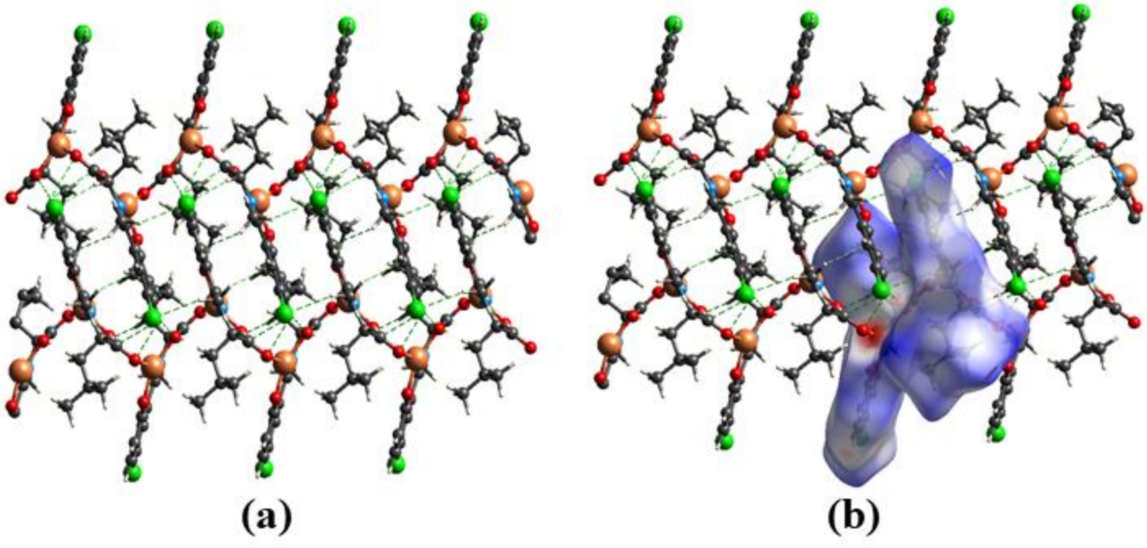

Figure S1. (a) Intermolecular hydrogen bonding interactions in CuL_{Cl}, (b) viewing the duplicate short contacts in Hirshfeld surface mapped with dnorm.

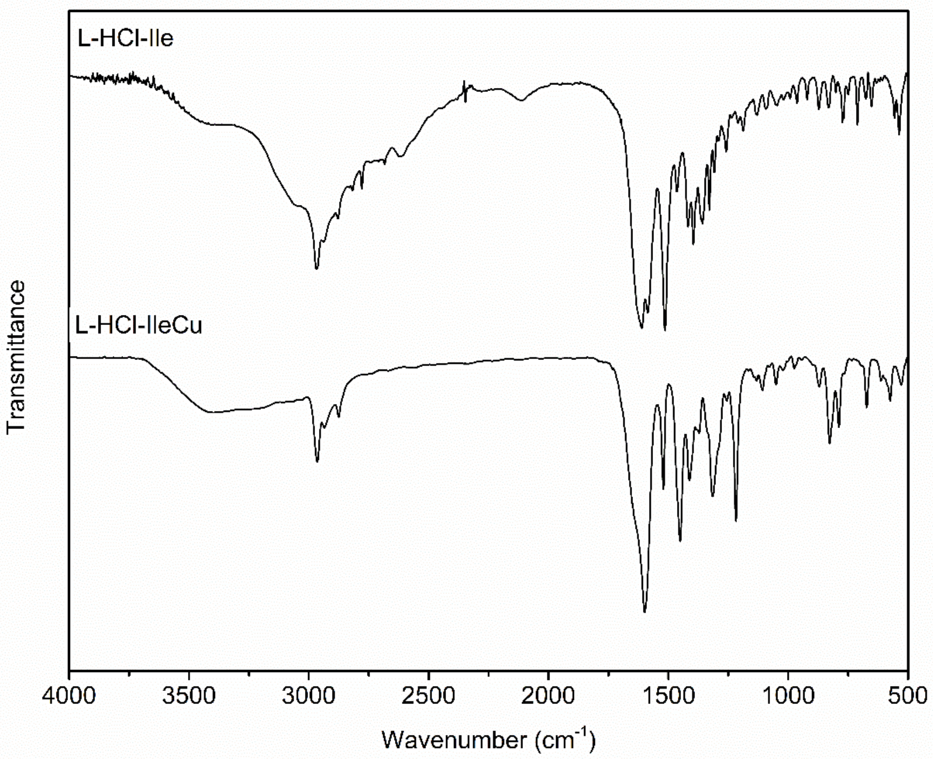

Figure S2. Infrared spectra of the ligand L_{Cl} and complex CuL_{Cl}.

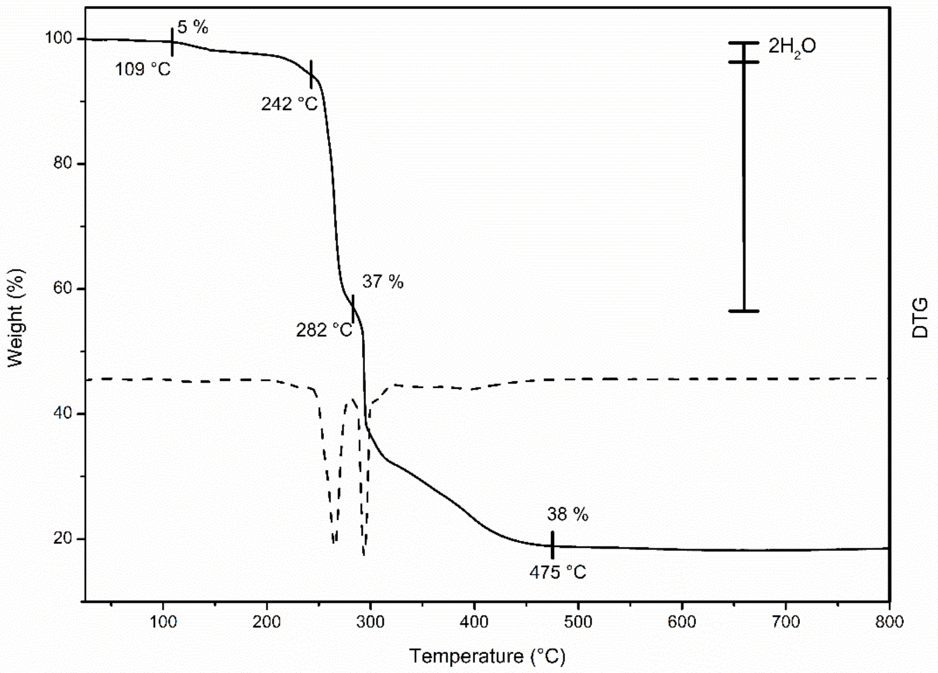

Figure S3. Thermogravimetric analysis (TG/DTG) of coordination polymer CuL_{Cl}.

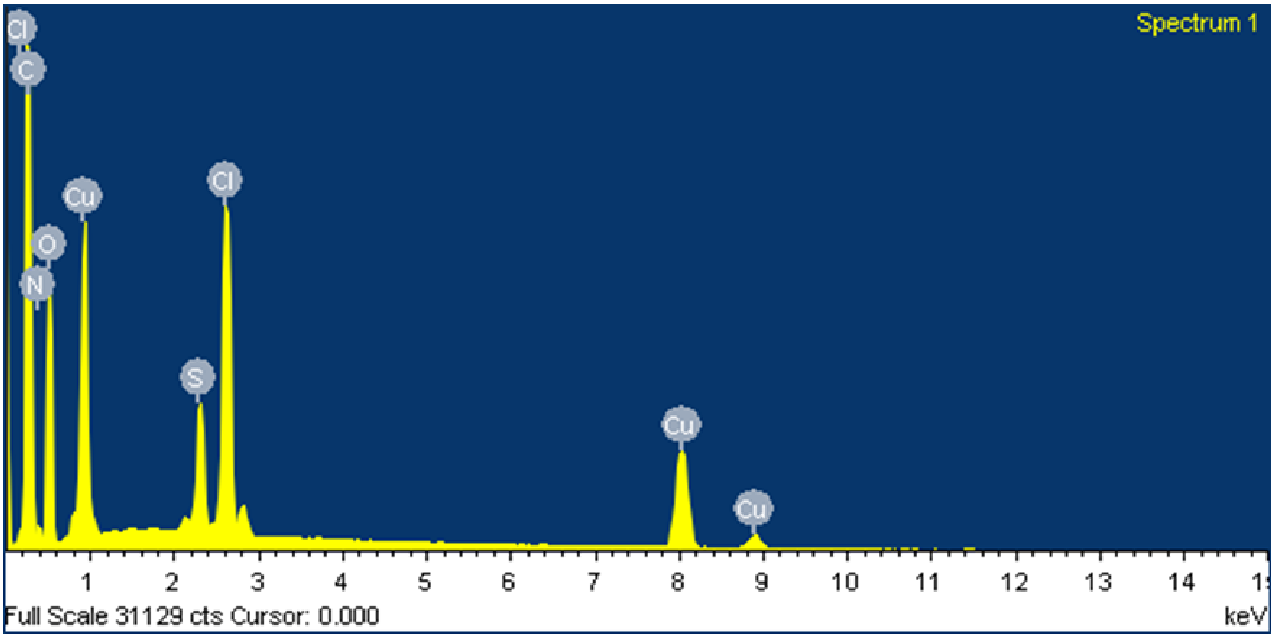

Figure S4. Representative EDX profile for the coordination polymer CuL_{Cl}.

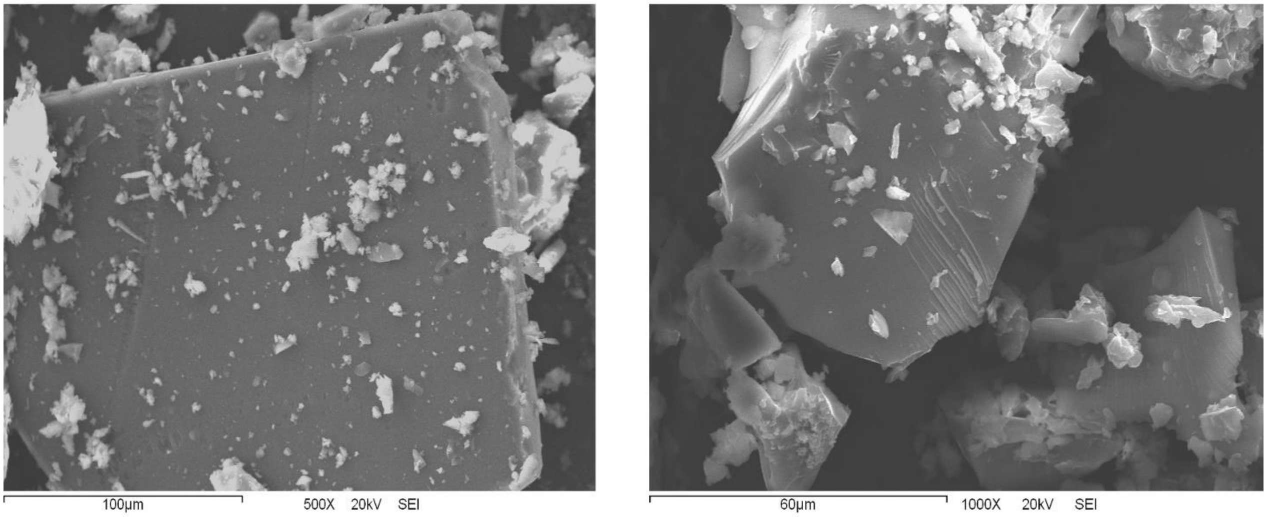

Figure S5. Representative different SEM images of the complex CuL_{Cl}.

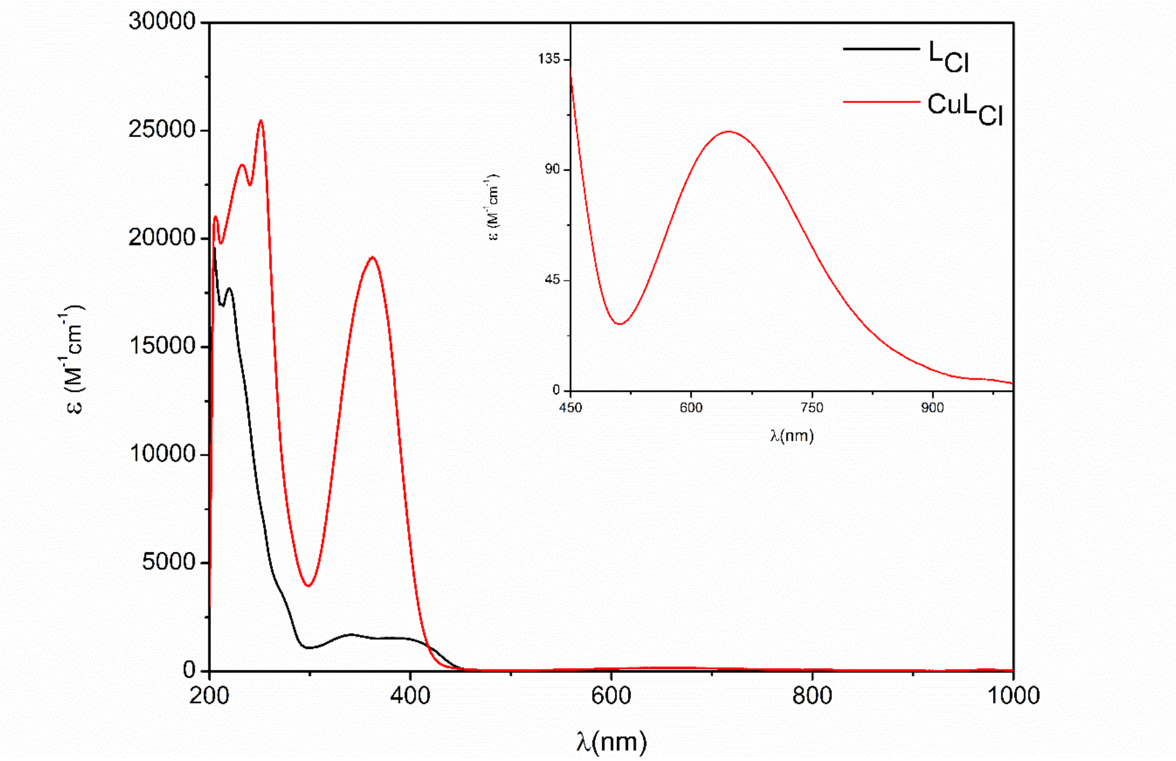

Figure S6. UV-Vis spectra of ligand and complex CuL$_{Cl}$.

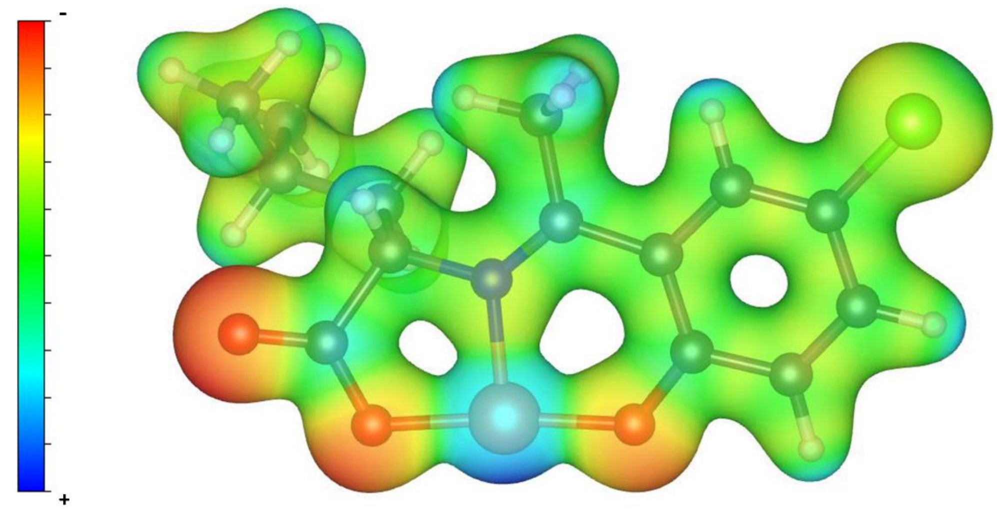

Figure S7. Map of molecular electrostatic potential (MESP). Regions in red indicate negative electrostatic potential (ESP), blue denotes positive ESP.

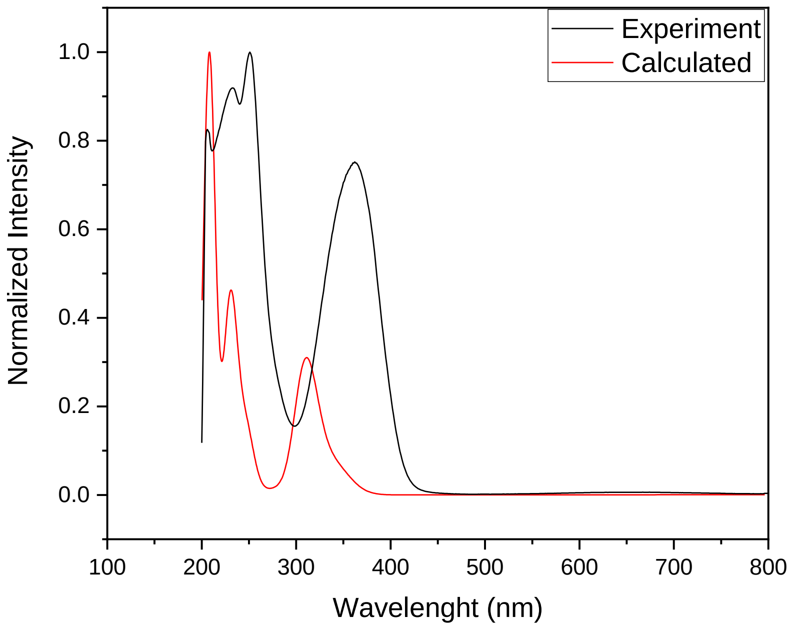

Figure S8. Comparison between experimental and calculated UV-vis spectrums

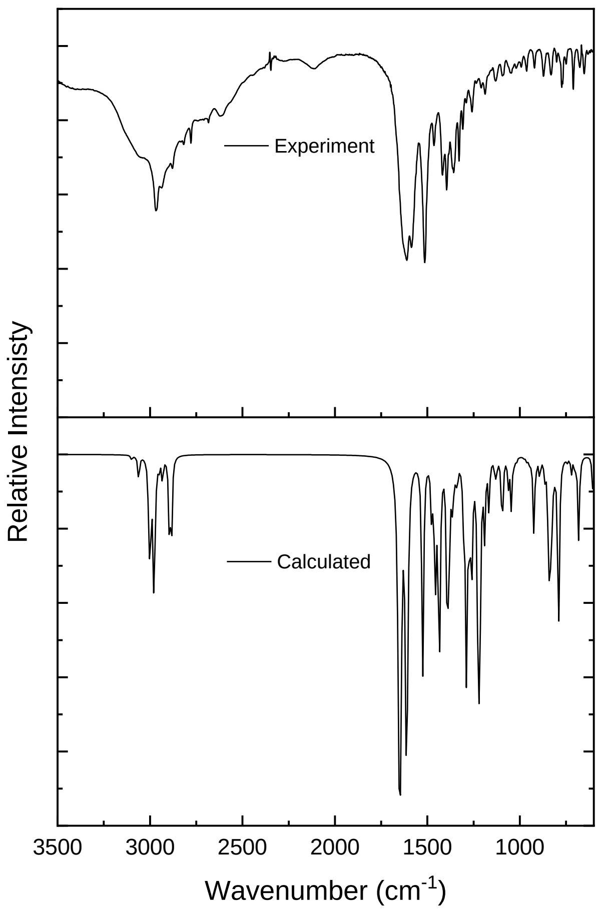

Figure S9. Experimental (top) and calculated with UM06-2X/LANL2DZ method (bottom) IR spectra.

Table S1. EDX analysis of weight (%) contribution of elements.

Complex	% C		% N		% O		% Cl		% Cu	
	**Calcd.**	**Obsd.**	**Calcd.**	**Obsd.**	**Calcd.**	**Obsd.**	**Calcd.**	**Obsd.**	**Calcd.**	**Obsd.**
**CuL$_{Cl}$**	48.51	52.03	4.10	4.94	17.49	18.05	10.24	10.11	18.35	19.21

Table S2. Natural Bond Orbital analysis. Donor and acceptor NBOs for interactions with Cu with indication of the second-order interaction energy $E^{(2)}$ and donor and acceptor orbitals for complex CuL$_{Cl}$.

**Donor**		**Acceptor**		**$E^{(2)}$ (kcal/mol)**
^{1}BD	C1-O1	^{3}LP*	Cu	2.52
BD	C2-N	LP*	Cu	1.44
BD	C5-O3	LP*	Cu	2.25
^{2}LP	N	LP*	Cu	18.78
LP	O1	LP*	Cu	26.55
LP	O3	LP*	Cu	29.17

BD stands for Bond, **LP** for Lone pair, and * indicates antibonding.

Table S3. Electronic transitions, their wavelength major contributions and their percentage calculated by TD-DFT M06-2X/LANL2DZ.

**No.**	**Wavelength (nm)**	**Osc. Strength**	**Major contribution**
**1**	1434.84	0	H-32(B)->LUMO(B) (29%), H-3(B)->LUMO(B) (11%), HOMO(B)->LUMO(B) (21%)
**2**	1068.37	0.0017	H-34(B)->LUMO(B) (10%), H-33(B)->LUMO(B) (12%), H-31(B)->LUMO(B) (18%)
**3**	761.01	0.0003	H-26(B)->LUMO(B) (53%)
**4**	730.09	0.0001	H-30(B)->LUMO(B) (10%), H-29(B)->LUMO(B) (56%)
**5**	472.46	0.0002	H-32(B)->LUMO(B) (11%), HOMO(B)->LUMO(B) (69%)
**6**	426.66	0	HOMO(A)->LUMO(A) (45%), HOMO(B)->L+1(B) (43%)
**7**	345.24	0.0226	H-4(B)->LUMO(B) (50%), H-1(B)->LUMO(B) (17%)
**8**	336.46	0.0166	HOMO(A)->LUMO(A) (13%), H-1(B)->LUMO(B) (53%), HOMO(B)->L+1(B) (16%)
**9**	333.83	0	H-2(A)->LUMO(A) (23%), H-2(B)->L+1(B) (21%)
**10**	319.57	0.0038	H-3(B)->LUMO(B) (45%), H-2(B)->LUMO(B) (30%)
**11**	310.27	0.186	HOMO(A)->LUMO(A) (22%), H-4(B)->LUMO(B) (32%), H-1(B)->LUMO(B) (16%), HOMO(B)->L+1(B) (22%)
**12**	290.50	0.0075	H-3(B)->LUMO(B) (21%), H-2(B)->LUMO(B) (52%)
**13**	289.77	0.0004	HOMO(A)->L+2(A) (15%), HOMO(A)->L+3(A) (15%), HOMO(B)->L+3(B) (11%), HOMO(B)->L+4(B) (17%)
**14**	277.13	0.0001	H-4(A)->LUMO(A) (48%), H-3(A)->LUMO(A) (18%)
**15**	274.82	0.0052	H-10(B)->LUMO(B) (68%)

**16**	266.71	0.0011	H-1(A)->L+7(A) (35%), H-1(B)->L+8(B) (28%)
**17**	249.75	0.0053	H-3(A)->L+7(A) (24%), H-3(B)->L+8(B) (31%)
**18**	248.35	0.049	H-11(B)->LUMO(B) (18%), H-2(B)->L+1(B) (13%)
**19**	245.03	0.0302	H-2(A)->LUMO(A) (13%), H-1(B)->L+1(B) (11%)
**20**	243.79	0.0066	H-1(A)->L+7(A) (30%), H-1(B)->L+8(B) (35%)
**21**	232.17	0.0111	HOMO(A)->L+1(A) (80%)
**22**	231.58	0.1805	H-1(A)->LUMO(A) (22%), H-11(B)->LUMO(B) (10%), H-4(B)->L+1(B) (12%), H-1(B)->L+1(B) (22%)
**23**	229.78	0.0599	H-4(B)->L+1(B) (43%), H-2(B)->L+1(B) (12%)
**24**	225.92	0.0085	H-2(A)->LUMO(A) (10%), H-1(A)->LUMO(A) (20%), H-1(B)->L+1(B) (21%)
**25**	223.86	0.039	H-2(A)->LUMO(A) (18%), H-1(A)->LUMO(A) (13%), H-2(B)->L+1(B) (22%), H-1(B)->L+1(B) (17%)
**26**	220.33	0.0014	HOMO(B)->L+2(B) (25%)
**27**	220.21	0.0008	H-5(B)->LUMO(B) (39%)
**28**	219.1269	0.0052	H-11(B)->LUMO(B) (15%), H-7(B)->LUMO(B) (23%)
**29**	216.2716	0.001	H-5(B)->LUMO(B) (18%), HOMO(B)->L+2(B) (16%), HOMO(B)->L+3(B) (14%), HOMO(B)->L+9(B) (10%)
**30**	213.9835	0.0004	HOMO(A)->L+8(A) (31%), HOMO(B)->L+9(B) (30%)
**31**	209.6169	0.0191	H-11(B)->L+1(B) (31%)
**32**	209.1572	0.333	HOMO(A)->L+2(A) (10%), HOMO(B)->L+4(B) (19%)
**33**	208.3978	0.151	H-2(A)->L+2(A) (11%), HOMO(A)->L+3(A) (11%)
**34**	205.3364	0.0463	H-3(B)->L+1(B) (38%)
**35**	203.9046	0.0066	H-4(A)->LUMO(A) (10%), H-3(A)->LUMO(A) (24%), H-3(B)->L+1(B) (17%)
**36**	202.7476	0.1272	H-3(A)->LUMO(A) (18%), HOMO(A)->L+2(A) (11%), H-3(B)->L+1(B) (13%)
**37**	200.3979	0.0072	H-5(A)->LUMO(A) (23%), H-11(B)->L+1(B) (10%)
**38**	199.8359	0	H-6(A)->LUMO(A) (22%), H-5(B)->L+1(B) (15%)
**39**	198.9253	0.0006	H-9(B)->LUMO(B) (54%), H-7(B)->LUMO(B) (27%)
**40**	198.594	0.0013	HOMO(A)->L+8(A) (42%), HOMO(B)->L+9(B) (40%)

Table S4. Experimental IR, and the theoretical frequencies (ω, cm^{-1}), infrared intensities (I_{IR}, km mol^{-1}), for the ---- complex, in the range 3900–1080 cm^{-1}

No.	ω_{exp}	ω	ω_{scaled}	I_{IR}	Vibrational mode	No.	ω_{exp}	ω	ω_{scaled}	I_{IR}	Vibrational mode
1		3269.21	3099.21	0.9694	υC-H_s (C6-H2, C7-H3, C9-H4)	27	1395	1509.58	1431.09	132.520	δ_{sc}C-H(C14-[H14, H16], C13-[H11, H13], C11-[H8, H9], C10-[H5, H7]), ρ_wC-H (C9-H4, C6-H2, C7-H3)
2		3266.47	3096.62	1.022	υC-H_a (C6-H2, C7-H3, C9-H4)	28		1502.79	1424.65	5.118	δ_{sc}C-H(C14-[H14, H16], C13-[H11, H13], C11-[H8, H9], C10-[H5, H7])
3		3248.38	3079.46	0.2383	υC-H_a (C6-H2, C7-H3)	29	1357	1462.94	1386.87	118.615	δ_{sc}C-H(C14-[H14, H16], C13-[H11, H13], C11-[H8, H9], C10-[H5, H7]), ρ_rC-C_s (C9-[C8,C4], C6-[C5,C7]) ρ_wC-H (C9-H4, C6-H2, C7-H3)
4		3227.59	3059.76	11.7391	υC-H_s (C10-[H5, H6, H7])						

5	2996	3168.94	3004.15	22.6612	υC-H$_a$ (C14-[H14, H15, H16])	30	1329	1449.91	1374.52	24.817	δ$_{sc}$C-H$_s$(C10-[H5, H6, H7], C13-[H11, H12, H13])
6		3166.83	3002.16	2.2796	υC-H$_a$ (C10-[H6, H7])	31		1449.25	1373.88	6.058	δ$_{sc}$C-H$_s$(C10-[H5, H6, H7], C13-[H11, H12, H13], C14-[H14, H15, H16])
7		3162.12	2997.76	46.5337	υC-H (C13-H12)$_a$ (C13-[H11, H13])$_s$	32		1432.34	1357.86	23.415	δ$_{sc}$C-H$_s$(C13-[H11, H12, H13], C14-[H14, H15, H16])
8	2967	3142.68	2979.26	59.4559	υC-H$_a$ (C14-[H15, H16], C2-H1, C11-[H8, H9],C13-H11, H13)	33		1417.495	1343.78	3.9045	ϱ$_w$C-H(C2-H1, C11-[H8, H9], C12-H10, C13-[H11, H12, H13], C14-[H14, H15, H16])
9		3137.00	2973.87	10.1878	υC-H (C2-H1, C14-H15)	34	1305	1407.37	1334.19	10.566	ϱ$_r$C-C$_a$ (C9-[C8,C4], C6-[C5,C7]), ϱ$_w$C-H(C2-H1, C10-[H5, H6, H7], C11-[H8, H9], C12-H10, C13-[H11, H12, H13], C14-[H14, H15, H16])
10		3133.01	2970.094	19.9733	υC-H$_a$(C14-[H15, H16], C2-H1, C13-[H11, H13])	35		1389.36	1317.12	0.147	ϱ$_w$C-H (C11-[H9], C12-H10)
11	2950	3111.85	2950.032	4.7014	υC-H (C2-H1, C12-H10, C14-[H14, H15, H16])$_s$ (C11-[H8, H9], C13-[H13, H11])$_a$	36		1370.97	1299.68	22.246	ϱ$_w$C-H(C2-H1, C11-[H8, H9], C12-H10, C9-H4, C6-H2, C7-H3)
12	2937	3092.51	2931.701	8.3972	υC-H$_a$ (C2-H1, C11-[H8, H9], C12-H10)	37	1288	1355.08	1284.62	144.424	ϱ$_w$C-H(C2-H1, C11-[H8, H9], C12-H10, C9-H4, C6-H2, C7-H3)
13		3088.83	2928.21	2.7077	υC-H$_s$ (C10-[H5, H6, H7])	38		1340.53	1270.82	31.824	ϱ$_w$C-H(C2-H1, C11-[H8, H9], C12-H10, C9-H4, C6-H2, C7-H3)
14	2896	3052.95	2894.193	32.9946	υC-H$_s$ (C13-[H11, H12, H13])	39	1257	1325.54	1256.61	69.489	ϱ$_w$C-H(C2-H1, C11-[H8, H9], C12-H10, C9-H4, C6-H2)
15	2879	3041.42	2883.263	24.5283	υC-H$_s$ (C14-[H14, H15, H16], C11-[H8, H9])	40		1313.50	1245.20	3.857	υC4-C9, ϱ$_w$C-H(C2-H1, C11-[H8, H9], C12-H10, C9-H4, C6-H2)
16		3040.31	2882.211	21.081	υC-H$_s$ (C14-[H14, H15, H16], C11-[H8, H9])	41	1235	1285.65	1218.79	171.9177	υC1-O1, ϱ$_w$C-H(C2-H1, C11-[H8, H9], C12-H10, C9-H4, C6-H2)
17	1612	1735.28	1645.042	602.4329	υC1=O2	42	1209	1277.58	1211.144	89.1625	δ$_{sc}$C-C(C4-[C3, C5]), ϱ$_r$C-C$_a$ (C9-[C4, C8]), ϱ$_w$C-H (C6-H2, C7-H3, C9-H4, C2-H1)

18	1583	1697.22	1608.968	276.0373	(υC3-N, υC5-O3, υC4-C9, υC6-C7)$_{s}$, ϱ$_{w}$C-H (C6-H2, C7-H3, C9-H4, C10-[H5, H7])	43	1187	1250.76	1185.718	35.3908	ϱ$_{w}$C-H(C2-H1, C11-[H8, H9], C12-H10, C13-[H11, H12, H13], C14-[H14, H15, H16])
19		1690.70	1602.781	56.6501	(υC3-N, υC8-C9, υC5-C6)$_{s}$, ϱ$_{w}$C-H (C6-H2, C7-H3, C9-H4, C10-[H5, H7])	44	1166	1225.90	1162.149	22.0773	ϱ$_{w}$C-H(C12-H10, C13-[H11, H12, H13], C14-[H14, H15, H16])
20	1511	1604.41	1520.983	146.0515	(υC3-N, υC4-C5, υC7-C8)$_{s}$, ϱ$_{w}$C-H (C6-H2, C7-H3, C9-H4, C10-[H5, H7])	45	1131	1189.24	1127.395	9.6468	ϱ$_{w}$C-H(C2-H1, C9-H4, C6-H2, C7-H3, C11-[H8, H9], C12-H10, C13-[H11, H12, H13], C14-[H14, H15, H16])
21	1464	1552.60	1471.867	30.0428	δ$_{sc}$C-H(C14-[H14, H15], C13-[H12, H13])	46		1178.42	1117.143	2.6579	ϱ$_{w}$C-H(C2-H1, C9-H4, C6-H2, C7-H3, C11-[H8, H9], C12-H10, C13-[H11, H12, H13], C14-[H14, H15, H16])
22	1416	1536.20	1456.318	12.7389	δ$_{sc}$C-H(C10-[H5, H7], C14-[H14, H15], C11-[H8, H9])	47		1152.97	1093.014	13.549	ϱ$_{w}$C-H(C2-H1, C9-H4, C6-H2, C7-H3, C10-[H5, H6, H7], C11-[H8, H9], C12-H10, C13-[H11, H12, H13], C14-[H14, H15, H16])
23		1532.32	1452.636	51.254	δ$_{sc}$C-H(C10-[H6, H7]), ϱ$_{w}$C-H (C9-H4)	48	1092	1148.10	1088.395	21.4036	υC8-Cl, ϱ$_{r}$C-C$_{a}$ (C7-[C6, C8]), ϱ$_{w}$C-H(C2-H1, C9-H4, C6-H2, C7-H3, C10-[H5, H6, H7],)
24		1528.50	1449.02	6.1033	δ$_{sc}$C-H(C14-[H15, H16], C13-[H11, H12])	49		1114.75	1056.784	12.3969	υC2-N, δ$_{sc}$C-C$_{s}$(C6-[C5, C7],C9-[C4, C8]), ϱ$_{w}$C-H (C6-H2, C7-H3, C9-H4, C10-[H5, H6, H7])
25		1524.88	1445.589	7.0945	δ$_{sc}$C-H(C14-[H15, H16], C13-[H12, H13])	50	1043	1097.92	1040.833	23.6133	υC2-N, δ$_{sc}$C-C$_{s}$(C6-[C5, C7],C9-[C4, C8]), ϱ$_{w}$C-H (C10-[H5, H6, H7])
26		1513.13	1434.45	8.326	δ$_{sc}$C-H(C14-[H15, H16], C13-[H11, H13], C11-[H8, H9], C10-[H5, H7]), ϱ$_{w}$C-H (C9-H4, C6-H2, C7-H3)	51		1081.99	1025.722	2.2395	ϱ$_{w}$C-H(C2-H1, C10-[H5, H6, H7], C11-[H8, H9], C12-H10, C13-[H11, H12, H13], C14-[H14, H15, H16])

*Abbreviations: υ, stretching; δ$_{sc,}$ scissoring; ϱw, wagging; ϱr, rocking; γ out-of-plane bending. Subscripts: a, asymmetric; s, symmetric.

Table S5. Experimental IR, and the theoretical frequencies (ω, cm^{-1}), infrared intensities (IIR, km mol^{-1}), for the ---- complex, in the range 1080-480 cm^{-1}

No.	ω_{exp}	ω	ω_{scaled}	I_{IR}	Vibrational mode
52	1017	1069.002	1013.414	2.2207	ϱ_wC-H(C2-H1, C10-[H5, H6, H7], C11-[H8, H9], C12-H10, C13-[H11, H12, H13], C14-[H14, H15, H16])
53		1024.349	971.0825	0.6472	γ(C6-H2, C7-H3)
54	962	1008.699	956.247	1.9294	δ_{sc}C-C(C6-[C5, C7],C9-[C4, C8], υN-Cu, ϱ_wC-H(C2-H1, C10-[H5, H6, H7], C11-[H8, H9], C12-H10, C13-[H11, H12, H13], C14-[H14, H15, H16])
55		996.7218	944.8923	1.3463	δ_{sc}C-C(C6-[C5, C7],C9-[C4, C8], υN-Cu, ϱ_wC-H(C2-H1, C10-[H5, H6, H7], C11-[H8, H9], C12-H10, C13-[H11, H12, H13], C14-[H14, H15, H16])
56		989.4396	937.9887	2.4926	ϱ_wC-H(C11-[H8, H9], C12-H10, C13-[H11, H12, H13], C14-[H14, H15, H16])
57	924	969.1427	918.7473	36.2884	δ_{sc}C2-C1-$O1_s$, ϱ_wC-H(C10-[H5, H6, H7], C11-[H8, H9], C12-H10, C13-[H11, H12, H13], C14-[H14, H15, H16])
58		956.6924	906.9444	3.1193	ϱ_wC-H(C11-[H8, H9], C12-H10, C13-[H11, H12, H13], C14-[H14, H15, H16])
59	894	935.2307	886.5987	8.5329	γC9-H4
60	869	906.3851	859.2531	7.6788	δ_{sc}C-C(C8-[C7, C9]) , υC3-C10, υO3-Cu, υC8-Cl
61	841	883.7278	837.774	60.6371	γC-H(C6-H2, C7-H3)
62		870.6632	825.3887	59.5712	δ_{sc}C-O(C1-[O1,O2], υO1-Cu, ϱ_wC-H(C11-[H8, H9], C12-H10, C13-[H11, H12, H13], C14-[H14, H15, H16])
63		854.3046	809.8808	7.351	υC-C(C12-[C11, C13, C14])
64	772	827.9208	784.8689	109.4205	υN-Cu, υO3-Cu, δ_{sc}C-C(C4-[C3, C5])
65		778.8587	738.358	1.8069	γC-H (C6-H2, C9-H4)
66		754.232	715.0119	7.0602	γC1-[O1, O2]
67		733.3749	695.2394	5.9983	δ_{sc}C1-$[O1, O2]_s$
68	711	712.6085	675.5529	40.9686	$(υN-Cu, υO3-Cu, υO1-Cu)_a$
69		697.7319	661.4498	0.0248	$(υN-Cu, υO3-Cu, υO1-Cu)_s$
70		638.1498	604.966	0.4674	γC3-C4, γC7-H3, γC3-N
71	649	630.3695	597.5903	18.7082	ϱ_wC6-[C5, C7], ϱ_wC5-O3
72	557	584.0815	553.7093	25.5506	ϱ_wC4-[C5, C9], ϱ_wC5-O3
73	537	545.3869	517.0268	20.9895	γC-C(C6-C5, C7-C8, C4-C3), γC3-N, γC5-O3
74		501.1339	475.0749	5.8158	γC-C(C6-C5, C7-C8, C4-C3), γC3-N
75		487.4654	462.1172	25.037	δ_{sc}C4-[C3, C5], ϱ_r (O1-Cu-O3)

*Abbreviations: υ, stretching; δ_{sc}, scissoring; ϱw, wagging; ϱr, rocking; γ out-of-plane bending. Subscripts: a, asymmetric; s, symmetric

Table S6. Atomic coordinates (x 10^4) and equivalent isotropic displacement parameters ($Å^2$x 10^3) for CuL_{Cl}. U(eq) is defined as one third of the trace of the orthogonalized U^{ij} tensor.

	x	y	z	U(eq)
C(1)	-85(5)	3211(3)	6959(2)	12(1)
C(2)	223(5)	3443(2)	6391(2)	13(1)
C(3)	915(5)	4232(2)	6222(2)	11(1)
C(4)	2122(5)	5566(2)	6437(2)	12(1)
C(5)	3171(5)	5869(2)	6945(2)	13(1)
C(6)	531(6)	6160(3)	6297(2)	17(1)
C(7)	1167(6)	6966(3)	6056(2)	21(1)
C(8)	-509(8)	7523(3)	6024(3)	47(2)

C(9)	2062(9)	6861(3)	5485(3)	46(2)
C(10)	1134(6)	4393(2)	5605(2)	14(1)
C(11)	-108(5)	2861(3)	5977(2)	13(1)
C(12)	-684(6)	2103(2)	6104(2)	13(1)
C(13)	-1003(6)	1862(2)	6651(2)	14(1)
C(14)	-696(6)	2414(2)	7066(2)	14(1)
C(15)	8141(5)	5490(3)	7997(2)	13(1)
C(16)	7015(5)	5751(2)	8501(2)	11(1)
C(17)	5800(6)	7072(2)	8745(2)	11(1)
C(18)	5130(5)	7873(2)	8596(2)	11(1)
C(19)	4845(5)	8128(3)	8036(2)	14(1)
C(20)	4260(6)	8936(2)	7948(2)	16(1)
C(21)	3936(6)	9465(3)	8372(2)	16(1)
C(22)	4190(6)	9195(2)	8915(2)	14(1)
C(23)	4760(5)	8430(3)	9025(2)	13(1)
C(24)	5983(6)	6879(2)	9359(2)	15(1)
C(25)	5411(5)	5141(3)	8592(2)	16(1)
C(26)	6016(6)	4300(3)	8765(2)	19(1)
C(27)	4311(8)	3745(3)	8769(2)	33(1)
C(28)	6962(8)	4315(3)	9335(2)	35(1)
Cl(1)	-1011(2)	1396(1)	5568(1)	18(1)
Cl(2)	3813(1)	9864(1)	9467(1)	19(1)
Cu(1)	5796(1)	6624(1)	7582(1)	12(1)
Cu(2)	824(1)	4746(1)	7373(1)	12(1)
N(1)	1324(4)	4776(2)	6586(1)	11(1)
N(2)	6233(4)	6549(2)	8370(1)	11(1)
O(1)	2592(4)	5646(2)	7417(1)	15(1)
O(2)	74(4)	3682(2)	7389(1)	16(1)
O(3)	4524(4)	6346(2)	6879(1)	15(1)
O(4)	7616(4)	5739(2)	7524(1)	14(1)
O(5)	9511(4)	5028(2)	8068(1)	14(1)
O(6)	5021(4)	7682(2)	7591(1)	15(1)

Table S7. Bond lengths [Å] and angles [°] for CuL_{Cl}.

C(1)-O(2)	1.308(5)
C(1)-C(14)	1.424(6)
C(1)-C(2)	1.440(6)
C(2)-C(11)	1.413(6)
C(2)-C(3)	1.465(6)
C(3)-N(1)	1.297(5)
C(3)-C(10)	1.521(5)
C(4)-N(1)	1.480(5)
C(4)-C(5)	1.525(6)
C(4)-C(6)	1.548(6)
C(4)-H(4)	1.0000
C(5)-O(3)	1.264(5)
C(5)-O(1)	1.269(5)
C(6)-C(7)	1.535(6)
C(6)-H(6A)	0.9900
C(6)-H(6B)	0.9900
C(7)-C(8)	1.520(7)
C(7)-C(9)	1.529(7)

C(7)-H(7)	1.0000
C(8)-H(8A)	0.9800
C(8)-H(8B)	0.9800
C(8)-H(8C)	0.9800
C(9)-H(9A)	0.9800
C(9)-H(9B)	0.9800
C(9)-H(9C)	0.9800
C(10)-H(10A)	0.9800
C(10)-H(10B)	0.9800
C(10)-H(10C)	0.9800
C(11)-C(12)	1.366(6)
C(11)-H(11)	0.9500
C(12)-C(13)	1.397(5)
C(12)-Cl(1)	1.767(4)
C(13)-C(14)	1.380(6)
C(13)-H(13)	0.9500
C(14)-H(14)	0.9500
C(15)-O(5)	1.259(5)
C(15)-O(4)	1.272(5)
C(15)-C(16)	1.524(5)
C(16)-N(2)	1.478(5)
C(16)-C(25)	1.550(5)
C(16)-H(16)	1.0000
C(17)-N(2)	1.295(5)
C(17)-C(18)	1.466(5)
C(17)-C(24)	1.521(5)
C(18)-C(23)	1.416(5)
C(18)-C(19)	1.432(6)
C(19)-O(6)	1.311(5)
C(19)-C(20)	1.429(6)
C(20)-C(21)	1.373(6)
C(20)-H(20)	0.9500
C(21)-C(22)	1.395(6)
C(21)-H(21)	0.9500
C(22)-C(23)	1.367(5)
C(22)-Cl(2)	1.759(4)
C(23)-H(23)	0.9500
C(24)-H(24A)	0.9800
C(24)-H(24B)	0.9800
C(24)-H(24C)	0.9800
C(25)-C(26)	1.528(6)
C(25)-H(25A)	0.9900
C(25)-H(25B)	0.9900
C(26)-C(27)	1.532(7)
C(26)-C(28)	1.534(6)
C(26)-H(26)	1.0000
C(27)-H(27A)	0.9800
C(27)-H(27B)	0.9800
C(27)-H(27C)	0.9800
C(28)-H(28A)	0.9800
C(28)-H(28B)	0.9800
C(28)-H(28C)	0.9800
Cu(1)-O(6)	1.852(3)

Cu(1)-N(2)	1.933(3)
Cu(1)-O(4)	1.974(3)
Cu(1)-O(3)	1.980(3)
Cu(2)-O(2)	1.855(3)
Cu(2)-N(1)	1.934(3)
Cu(2)-O(1)	1.967(3)
Cu(2)-O(5)#1	1.980(3)

O(2)-C(1)-C(14)	116.3(4)
O(2)-C(1)-C(2)	125.6(4)
C(14)-C(1)-C(2)	118.1(4)
C(11)-C(2)-C(1)	117.6(4)
C(11)-C(2)-C(3)	118.5(4)
C(1)-C(2)-C(3)	123.9(4)
N(1)-C(3)-C(2)	121.2(4)
N(1)-C(3)-C(10)	121.0(4)
C(2)-C(3)-C(10)	117.8(3)
N(1)-C(4)-C(5)	106.8(3)
N(1)-C(4)-C(6)	109.9(3)
C(5)-C(4)-C(6)	108.9(3)
N(1)-C(4)-H(4)	110.4
C(5)-C(4)-H(4)	110.4
C(6)-C(4)-H(4)	110.4
O(3)-C(5)-O(1)	123.3(4)
O(3)-C(5)-C(4)	119.0(4)
O(1)-C(5)-C(4)	117.7(3)
C(7)-C(6)-C(4)	115.2(3)
C(7)-C(6)-H(6A)	108.5
C(4)-C(6)-H(6A)	108.5
C(7)-C(6)-H(6B)	108.5
C(4)-C(6)-H(6B)	108.5
H(6A)-C(6)-H(6B)	107.5
C(8)-C(7)-C(9)	110.8(4)
C(8)-C(7)-C(6)	108.7(4)
C(9)-C(7)-C(6)	111.5(4)
C(8)-C(7)-H(7)	108.6
C(9)-C(7)-H(7)	108.6
C(6)-C(7)-H(7)	108.6
C(7)-C(8)-H(8A)	109.5
C(7)-C(8)-H(8B)	109.5
H(8A)-C(8)-H(8B)	109.5
C(7)-C(8)-H(8C)	109.5
H(8A)-C(8)-H(8C)	109.5
H(8B)-C(8)-H(8C)	109.5
C(7)-C(9)-H(9A)	109.5
C(7)-C(9)-H(9B)	109.5
H(9A)-C(9)-H(9B)	109.5
C(7)-C(9)-H(9C)	109.5
H(9A)-C(9)-H(9C)	109.5
H(9B)-C(9)-H(9C)	109.5
C(3)-C(10)-H(10A)	109.5
C(3)-C(10)-H(10B)	109.5
H(10A)-C(10)-H(10B)	109.5

C(3)-C(10)-H(10C)	109.5
H(10A)-C(10)-H(10C)	109.5
H(10B)-C(10)-H(10C)	109.5
C(12)-C(11)-C(2)	121.9(4)
C(12)-C(11)-H(11)	119.1
C(2)-C(11)-H(11)	119.1
C(11)-C(12)-C(13)	121.9(4)
C(11)-C(12)-Cl(1)	119.6(3)
C(13)-C(12)-Cl(1)	118.6(3)
C(14)-C(13)-C(12)	117.9(4)
C(14)-C(13)-H(13)	121.0
C(12)-C(13)-H(13)	121.0
C(13)-C(14)-C(1)	122.7(4)
C(13)-C(14)-H(14)	118.7
C(1)-C(14)-H(14)	118.7
O(5)-C(15)-O(4)	123.5(4)
O(5)-C(15)-C(16)	118.6(4)
O(4)-C(15)-C(16)	117.8(3)
N(2)-C(16)-C(15)	106.7(3)
N(2)-C(16)-C(25)	110.0(3)
C(15)-C(16)-C(25)	108.4(3)
N(2)-C(16)-H(16)	110.5
C(15)-C(16)-H(16)	110.5
C(25)-C(16)-H(16)	110.5
N(2)-C(17)-C(18)	121.4(3)
N(2)-C(17)-C(24)	121.2(3)
C(18)-C(17)-C(24)	117.4(3)
C(23)-C(18)-C(19)	118.0(4)
C(23)-C(18)-C(17)	118.7(4)
C(19)-C(18)-C(17)	123.4(4)
O(6)-C(19)-C(20)	116.2(4)
O(6)-C(19)-C(18)	126.2(4)
C(20)-C(19)-C(18)	117.6(4)
C(21)-C(20)-C(19)	123.0(4)
C(21)-C(20)-H(20)	118.5
C(19)-C(20)-H(20)	118.5
C(20)-C(21)-C(22)	118.1(4)
C(20)-C(21)-H(21)	120.9
C(22)-C(21)-H(21)	120.9
C(23)-C(22)-C(21)	121.5(4)
C(23)-C(22)-Cl(2)	119.4(3)
C(21)-C(22)-Cl(2)	119.1(3)
C(22)-C(23)-C(18)	121.8(4)
C(22)-C(23)-H(23)	119.1
C(18)-C(23)-H(23)	119.1
C(17)-C(24)-H(24A)	109.5
C(17)-C(24)-H(24B)	109.5
H(24A)-C(24)-H(24B)	109.5
C(17)-C(24)-H(24C)	109.5
H(24A)-C(24)-H(24C)	109.5
H(24B)-C(24)-H(24C)	109.5
C(26)-C(25)-C(16)	115.6(3)
C(26)-C(25)-H(25A)	108.4

C(16)-C(25)-H(25A)	108.4
C(26)-C(25)-H(25B)	108.4
C(16)-C(25)-H(25B)	108.4
H(25A)-C(25)-H(25B)	107.4
C(25)-C(26)-C(27)	109.4(4)
C(25)-C(26)-C(28)	110.8(4)
C(27)-C(26)-C(28)	110.8(4)
C(25)-C(26)-H(26)	108.6
C(27)-C(26)-H(26)	108.6
C(28)-C(26)-H(26)	108.6
C(26)-C(27)-H(27A)	109.5
C(26)-C(27)-H(27B)	109.5
H(27A)-C(27)-H(27B)	109.5
C(26)-C(27)-H(27C)	109.5
H(27A)-C(27)-H(27C)	109.5
H(27B)-C(27)-H(27C)	109.5
C(26)-C(28)-H(28A)	109.5
C(26)-C(28)-H(28B)	109.5
H(28A)-C(28)-H(28B)	109.5
C(26)-C(28)-H(28C)	109.5
H(28A)-C(28)-H(28C)	109.5
H(28B)-C(28)-H(28C)	109.5
O(6)-Cu(1)-N(2)	95.59(13)
O(6)-Cu(1)-O(4)	155.81(12)
N(2)-Cu(1)-O(4)	85.08(13)
O(6)-Cu(1)-O(3)	95.51(12)
N(2)-Cu(1)-O(3)	154.56(13)
O(4)-Cu(1)-O(3)	93.92(12)
O(2)-Cu(2)-N(1)	95.71(14)
O(2)-Cu(2)-O(1)	156.32(12)
N(1)-Cu(2)-O(1)	85.07(13)
O(2)-Cu(2)-O(5)#1	94.14(12)
N(1)-Cu(2)-O(5)#1	156.07(13)
O(1)-Cu(2)-O(5)#1	94.47(12)
C(3)-N(1)-C(4)	123.1(3)
C(3)-N(1)-Cu(2)	127.2(3)
C(4)-N(1)-Cu(2)	109.5(3)
C(17)-N(2)-C(16)	123.2(3)
C(17)-N(2)-Cu(1)	127.3(3)
C(16)-N(2)-Cu(1)	109.3(2)
C(5)-O(1)-Cu(2)	112.7(3)
C(1)-O(2)-Cu(2)	125.7(3)
C(5)-O(3)-Cu(1)	113.0(3)
C(15)-O(4)-Cu(1)	112.1(3)
C(15)-O(5)-Cu(2)#2	113.6(3)
C(19)-O(6)-Cu(1)	125.5(3)

Symmetry transformations used to generate equivalent atoms:
#1 x-1,y,z #2 x+1,y,z

Table S8. Anisotropic displacement parameters (Å^{2}x 10^3)for CuL$_{Cl}$. The anisotropic displacement factor exponent takes the form: -2p^2[h^{2}a*^{2}U^{11} + ... + 2 h k a* b* U^{12}]

	U^{11}	U^{22}	U^{33}	U^{23}	U^{13}	U^{12}
C(1)	11(2)	15(2)	10(2)	-3(2)	2(2)	5(2)
C(2)	10(2)	13(2)	14(2)	-2(2)	-2(2)	3(2)
C(3)	8(2)	14(2)	12(2)	2(2)	-1(2)	2(2)
C(4)	13(2)	9(2)	13(2)	-1(2)	-1(2)	1(2)
C(5)	13(2)	10(2)	15(2)	-2(2)	-2(2)	4(2)
C(6)	15(2)	18(2)	17(2)	2(2)	-4(2)	1(2)
C(7)	22(2)	15(2)	26(2)	3(2)	-3(2)	2(2)
C(8)	41(3)	28(3)	72(4)	21(3)	2(3)	11(3)
C(9)	74(4)	24(3)	40(3)	13(3)	15(3)	3(3)
C(10)	19(2)	14(2)	10(2)	-1(2)	-3(2)	-4(2)
C(11)	12(2)	15(2)	11(2)	1(2)	-1(2)	4(2)
C(12)	11(2)	12(2)	15(2)	-4(2)	-2(2)	2(2)
C(13)	12(2)	7(2)	22(2)	2(2)	-2(2)	2(2)
C(14)	15(2)	13(2)	14(2)	2(2)	3(2)	3(2)
C(15)	14(2)	11(2)	12(2)	-1(2)	1(2)	-5(2)
C(16)	13(2)	9(2)	12(2)	-2(2)	2(2)	-1(2)
C(17)	9(2)	12(2)	11(2)	1(2)	1(2)	-4(2)
C(18)	11(2)	9(2)	13(2)	0(2)	-1(2)	-3(2)
C(19)	11(2)	15(2)	16(2)	2(2)	0(2)	-4(2)
C(20)	18(2)	17(2)	13(2)	3(2)	-4(2)	-3(2)
C(21)	14(2)	12(2)	21(2)	4(2)	-1(2)	0(2)
C(22)	12(2)	11(2)	19(2)	-6(2)	1(2)	-2(2)
C(23)	13(2)	16(2)	11(2)	-1(2)	-1(2)	-3(2)
C(24)	19(2)	14(2)	12(2)	1(2)	4(2)	3(2)
C(25)	14(2)	14(2)	20(2)	0(2)	3(2)	-3(2)
C(26)	21(2)	15(2)	22(2)	0(2)	7(2)	-2(2)
C(27)	39(3)	18(3)	42(3)	2(2)	2(3)	-11(3)
C(28)	47(3)	22(3)	35(3)	14(2)	-11(3)	-4(2)
Cl(1)	23(1)	13(1)	19(1)	-5(1)	-3(1)	-1(1)
Cl(2)	22(1)	15(1)	22(1)	-5(1)	2(1)	3(1)
Cu(1)	15(1)	13(1)	9(1)	-2(1)	-1(1)	-1(1)
Cu(2)	14(1)	12(1)	9(1)	-2(1)	1(1)	0(1)
N(1)	10(2)	10(2)	12(2)	0(1)	1(1)	0(1)
N(2)	13(2)	10(2)	9(2)	-1(1)	1(1)	0(1)
O(1)	17(1)	18(2)	11(1)	-2(1)	0(1)	-1(1)
O(2)	21(1)	15(2)	12(1)	-2(1)	3(1)	-1(1)
O(3)	15(1)	16(2)	14(1)	-2(1)	-1(1)	-3(1)
O(4)	16(1)	17(2)	10(1)	-3(1)	0(1)	-1(1)
O(5)	15(1)	13(2)	13(1)	-1(1)	3(1)	2(1)
O(6)	22(1)	16(2)	8(1)	2(1)	-2(1)	1(1)
